# Optimization of Lead Placement in the Right Ventricle During Cardiac Resynchronization Therapy. A Simulation Study

**DOI:** 10.3389/fphys.2019.00074

**Published:** 2019-02-11

**Authors:** Edison F. Carpio, Juan F. Gomez, Rafael Sebastian, Alejandro Lopez-Perez, Eduardo Castellanos, Jesus Almendral, Jose M. Ferrero, Beatriz Trenor

**Affiliations:** ^1^Centre for Research and Innovation in Bioengineering (Ci2B), Universitat Politècnica de València, Valencia, Spain; ^2^Computational Multiscale Simulation Lab (CoMMLab), Department of Computer Science, Universitat de València, Valencia, Spain; ^3^Electrophysiology Laboratory and Arrhythmia Unit, Grupo HM Hospitales, Hospital Monteprincipe, University CEU-San Pablo, Madrid, Spain

**Keywords:** cardiac resynchronization therapy, heart failure, LBBB, computational modeling, QRS duration, optimization

## Abstract

Patients suffering from heart failure and left bundle branch block show electrical ventricular dyssynchrony causing an abnormal blood pumping. Cardiac resynchronization therapy (CRT) is recommended for these patients. Patients with positive therapy response normally present QRS shortening and an increased left ventricle (LV) ejection fraction. However, around one third do not respond favorably. Therefore, optimal location of pacing leads, timing delays between leads and/or choosing related biomarkers is crucial to achieve the best possible degree of ventricular synchrony during CRT application. In this study, computational modeling is used to predict the optimal location and delay of pacing leads to improve CRT response. We use a 3D electrophysiological computational model of the heart and torso to get insight into the changes in the activation patterns obtained when the heart is paced from different regions and for different atrioventricular and interventricular delays. The model represents a heart with left bundle branch block and heart failure, and allows a detailed and accurate analysis of the electrical changes observed simultaneously in the myocardium and in the QRS complex computed in the precordial leads. Computational simulations were performed using a modified version of the O'Hara et al. action potential model, the most recent mathematical model developed for human ventricular electrophysiology. The optimal location for the pacing leads was determined by QRS maximal reduction. Additionally, the influence of Purkinje system on CRT response was assessed and correlation analysis between several parameters of the QRS was made. Simulation results showed that the right ventricle (RV) upper septum near the outflow tract is an alternative location to the RV apical lead. Furthermore, LV endocardial pacing provided better results as compared to epicardial stimulation. Finally, the time to reach the 90% of the QRS area was a good predictor of the instant at which 90% of the ventricular tissue was activated. Thus, the time to reach the 90% of the QRS area is suggested as an additional index to assess CRT effectiveness to improve biventricular synchrony.

## Introduction

Heart failure (HF) constitutes a major public health problem worldwide and much attention has been paid to the understanding of the arrhythmogenic mechanisms in the failing heart induced by the structural, electrical, and metabolic remodeling. Heart failure is also characterized by a compromised ventricular contraction, which is fundamental for an optimal cardiac function. Lack of synchrony in heart contraction is worsened when the failing heart is also affected by left bundle branch block (LBBB). These patients present electrical and mechanical ventricular dyssynchrony causing pump dysfunction, reduced functional capacity, and myocardial remodeling. In particular, LBBB is associated with delayed contraction of the left ventricle (LV), reduced ventricular performance and widening of the QRS complex.

The relative QRS duration (QRSd) provides a powerful prognostic value for patients with HF and is a primary indicator of eligibility for cardiac resynchronization therapy (CRT). CRT helps to reduce mortality and morbidity associated with HF (Abraham et al., 2002; Cleland et al., 2005). Recent studies have also concluded that patients with LBBB are more likely to respond to CRT than those with right bundle branch block (RBBB) or nonspecific interventricular conduction delays (IVCDs) (Zareba et al., 2011).

During CRT, two synchronized electrical stimuli are usually delivered to reduce ventricular dyssynchrony. One stimulation lead is usually placed on the apex of the right ventricle (RV), and the other one on the epicardium of the LV lateral wall. Patients with positive therapy response present QRS shortening and an increased LV ejection fraction (LVEF) (Bonakdar et al., 2009; Zhang et al., 2015; Coppola et al., 2016). However, around one third of the patients do not respond favorably to this therapy (Linde et al., 2012; Bertaglia et al., 2017) and implantation issues, such as perforation of the RV apex, have been observed.

Optimal location of pacing leads is crucial to achieve the best degree of ventricular synchrony. LV lead position has been recognized as an important determinant for response to CRT since the initial development of this therapy (Singh et al., 2011; Crozier et al., 2016; Lee et al., 2017). Experimental studies and computational models (Lopez-Perez et al., 2015) have been used to optimize LV lead location. In current guidelines (Brignole et al., 2013), the LV posterior-lateral wall is the recommended LV region for CRT application. Several studies have reported the beneficial results of pacing from the lateral region of the LV (Rossillo et al., 2004). However, there are still several open questions.

First, as suggested by Zanon et al. the ideal LV lead placement should be the latest electrical intrinsic activated region (Zanon et al., 2014), typically the postero-lateral wall (Sipal et al., 2018). This location provided the maximum increase in contractility, expressed as the highest value of the first derivative of LV pressure over time (LV *dP/dt*_max_). However, the electromechanical modeling study by Pluijmert et al. (2016) determined that in fascicular block conditions the latest activated area did not provide the maximum response in contractility. A different criterion suggested in the literature is to place the LV lead in the site corresponding to the shortest QRS registered. Nevertheless, simulation studies that apply this last non-invasive criterion (Miri et al., 2009a,b) estimated QRSd through calculation of the total ventricular activation time (TAT), a parameter not easily accessible in clinical or even experimental settings. In addition, studies such as Potse et al. (2012) have observed that biventricular pacing did not change QRS duration but reduced total ventricular activation time when the stimulation was applied in one point of the LV free wall.

Second, there is controversy about whether a higher degree of synchrony can be achieved by stimulating from points in the RV other than the apex (Da Costa et al., 2013; Pastore et al., 2013). Third, individualized programming of the atrioventricular delay (AVD) and interventricular delay (VVD) intervals is not typically performed in most patients in the normal clinical practice, and it has been primarily reserved for non CRT responders (Gras et al., 2009). The largest trials studying CRT used various methods to optimize these intervals, most frequently based on echocardiography and intracardiac electrogram interval measurements, but unequivocal proof of the benefit brought by optimization is still lacking (Abraham et al., 2010; Krum et al., 2012; Brugada et al., 2014). Echocardiography presents inherent variability of results and is highly operator dependent. Optimization based on intracardiac electrogram intervals has not proved yet to be of clear benefit above arbitrary atrioventricular interval (Ulč and Vančura, 2013). Another optimization method based on the surface ECG uses fusion with intrinsic conduction and avoids echocardiographic atrioventricular and biventricular optimization (Arbelo et al., 2014). Applying this method Arbelo et al. determined that electrocardiographic optimization improved invasive LV *dP/dt*_max._ Similarly, randomized studies demonstrated that electrocardiographic optimization had superior LV remodeling at 6-month follow up although survival was not different, compared with optimization by echocardiography (Bertini et al., 2008; Tamborero et al., 2011). All these results suggest that minimizing QRSd could be used as a non-invasive method to optimize CRT.

In this study, we used a 3D biophysical model of the heart and torso to optimize pacing leads location, AVD, and VVD settings during CRT procedure, based on the shortest QRS duration measured on the torso surface. Results were compared with other optimization criteria. This analysis was used to define an electrical biomarker that relates the optimal lead configuration with the observed surface electrocardiogram signals.

## Methods

### Anatomical Model

A 3D biventricular model of the heart was built from segmentation of a DE-MRI images stack. The cardiac DE-MRI was acquired from the Hospital Clinic Universitari de Valencia (Valencia, Spain). Regarding the ethical considerations, the protocol was approved by the Ethics Committee for Clinical Research of the Hospital Clinic Universitari de Valencia, which certifies that the present study was conducted in accordance with the recommendations gathered in the Declaration of Helsinki, originally adopted by the General Assembly of the World Medical Association in 1964, and in its subsequent revisions. Furthermore, the patient, who underwent the standard clinical protocol, gave written informed consent for the use of his anonymized clinical data in this study.

**Figure 1:**
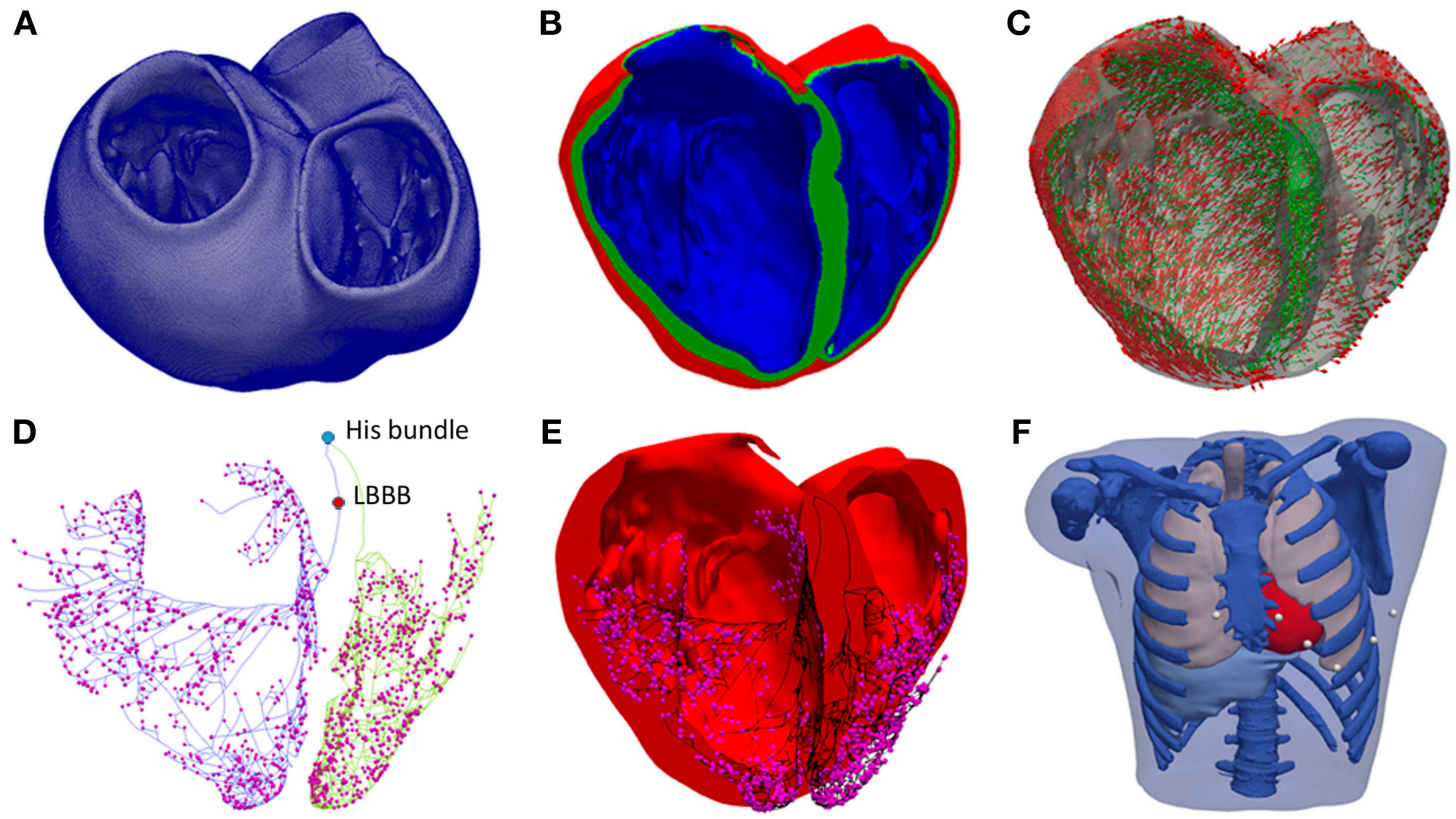
Anatomical model. **(A)** Biventricular hexahedral mesh of a segmented human heart. **(B)** Model color-coded to show the assignment of the elements to the different cellular model in order to model the transmural heterogeneity: endocardial cells (blue), midmyocardial cells (green) and epicardial cells (red). **(C)** Arrows indicating the principal myofiber orientation of epicardial (red) and midmyocardial (green) cells. **(D)** Purkinje System (PS), including three main LV branches (posterior, septal, anterior) and RV main brunches (septal and anterior). Purkinje-Junctions are represented as magenta spheres. His Bundle, and the location of the LBBB are labeled in the model. **(E)** PS (black) coupled to the biventricular model. **(F)** Torso model with the biventricular mesh embedded (red) and precordial leads location (white).

Manual image segmentation was performed using Seg3D software (Scientific Computing and Imaging Institute, University of Utah, USA) (Seg3D, 2013), including papillary muscles and main endocardial trabeculations (Figure 1A). From the segmented DE-MRI stack, a surface model of the ventricles was generated and subsequently meshed using MeshGems-Hexa (Distene S.A.S., Bruyeres-le-Chatel, France), obtaining a hexahedra-based volume mesh comprised of 4 million nodes (vertices) and 3.71 million elements, with an average edge length of 0.4 mm (see Supplementary Material for further detailed information). Transmural heterogeneity (Figure 1B) was defined by three different transmural layers for endocardial (blue), midmyocardial (green), and epicardial (red) cells within the volume mesh of our ventricular model, spanning 17, 41, and 42% of ventricular wall thickness, respectively (Sicouri and Antzelevitch, 1991; Sicouri et al., 1994; Desk et al., 1998).

To include the anisotropy of the cardiac muscle through fibers orientation (Figure 1C), we implemented Streeter's rule-based method (Streeter, 1979) modeled by the set of equations described in Sebastian et al. (2009) defining the helix (α_h_) and transmural (α_t_) angles. In papillary muscles and endocardial trabeculations, fibers are known to be aligned parallel to the longitudinal axis of those anatomical structures (Greenbaum et al., 1981). In order to reproduce such configuration, we performed the topological skeletonization of the volume mesh to extract the medial axes of each one of those structures, what enabled to properly assign the fiber orientation. Finally, we performed a Gaussian smoothing with a 3D kernel to soften abrupt transitions in fibers direction between the myocardial wall and the papillary muscles and trabeculations.

A Purkinje system (PS) network (Figures 1D,E) was developed based on a stochastic grown method (Sebastian et al., 2013) formed by linear elements. The RV section was composed of two main branches, one descending to the apex, and another extending to the surroundings of the moderator band, with several subdivisions. The LV section was formed by three main branches with several subdivisions: one descending to the apex toward the papillary muscles of the lateral wall, another one to the anterior wall, and the last one to the posterior wall. The location of the PMJs that start the endocardial activation from the main PS branches was optimized to obtain a typical ECG wave morphology in the precordial leads. Purkinje-Myocardial junctions (PMJs) conductivity were adjusted to allow retrograde and anterograde electrical propagation. A total of 1391 PMJ were distributed across the RV and LV.

The biventricular mesh was fit into a human torso mesh (Ferrer et al., 2015) to be able to properly solve the forward problem in electrophysiology and simulate the electrocardiogram (ECG) (Figure 1F). The torso dataset was obtained from the online open repository at the Center for Integrative Biomedical Computing (CBIC) from University of Utah (MacLeod et al., 1991). The torso volume mesh was made of tetrahedral elements of 0.5 mm spatial resolution. Note that the problem of passive propagation of extracellular potentials, i.e., only diffusion without reaction component, does not require such a fine spatial resolution outside the heart domain (Prassl et al., 2009); for this reason, the torso mesh is highly refined only in the region where it intersects with the ventricles (see Supplementary Material for complementary description).

### Electrophysiological Model

O'Hara et al. (2011) model is the most recent action potential model developed for human ventricular electrophysiology. Our simulations were conducted using a modified version of this model to achieve realistic conduction velocity and electrical propagation in 3D ventricular tissue. For this reason, the original fast sodium current (I_Na_) formulation was modified. Firstly, the steady state inactivation (h_ss_ and j_ss_) and activation (m_ss_) gates were changed as in Passini et al. (2016) and Mora et al. (2017), respectively. Secondly, the time constant of the inactivation gates was modified as in Dutta et al. (2017). Finally, the sodium conductance (G_Na_) was decreased to 23% of its original value to obtain approximately a maximum upstroke velocity (dV/dt_max_) of 260 V/ms as in the original O'Hara et al. (2011) model. Furthermore, the late sodium current (I_NaL_) conductance (G_NaL_) was duplicated to maintain the relationship between I_NaL_ and peak I_Na_ observed in voltage-clamp experiments as described in Mora et al. (2017). All these changes are detailed in the Supplementary Material together with the action potential (Figure S1) obtained with the original and modified O'Hara et al. models. The action potential model for Purkinje cells developed by Stewart et al. (2009) was used in the cardiac conduction system.

The electrical propagation through the ventricles was calculated by solving the monodomain equation (Equation 1) using ELVIRA FEM software (Heidenreich et al., 2010),

(1) $$\begin{array}{r} \nabla \cdot \left(\text{D}\nabla V_{m}\right)=C_{m}\frac{\partial V_{m}}{\partial t}+I_{ion}+I_{stim} \end{array}$$

where **D** is the equivalent conductivity tensor, *V*_m_ the transmembrane potential field, *C*_m_ the cell membrane capacitance, *I*_ion_ the transmembrane ionic current and *I*_stim_ the transmembrane stimulation current.

The ECG was simulated by solving the extracellular potential φ_e_ from the equation

(2) $$\begin{array}{r} \nabla \cdot \left(\left[D_{i}+D_{e}\right]\nabla \varphi_{e}\right)=-\nabla \cdot \left(D_{i}\nabla V_{m}\right) \end{array}$$

where *D*_i_ and *D*_e_ are the volume-average conductivity tensors of the intra and extracellular domains, respectively (Potse et al., 2006). The reaction-diffusion simulation was run on the biventricular mesh. The right-hand side of Equation (2) was evaluated on this fine mesh and then interpolated on the coarser torso mesh. The extracellular potential φ_e_ was solved on the coarser mesh. The precordial ECG leads were then computed by extracting the extracellular potential at the electrode locations taking into account the Wilson terminal, as in clinical practice (see Supplementary Material).

In order to establish the conductivities that will define the conduction velocities (CV) in the heart domain, we performed a set of test simulations on a 3D slab model (20 × 20 × 6 mm) composed of regular hexahedral elements (voxels) with an edge length of 0.4 mm, matching the average length in the ventricular model. As a result, we set the conductivity values to 0.5 S/m and 0.1 S/m for longitudinal (σ_L_) and transversal (σ_T_) conductivity, respectively. This resulted in a CV of 0.61 m/s along the fiber direction and of 0.29 m/s in transverse direction. These values are consistent with experimental measurements in human ventricles (Taggart et al., 2000).

CV in the PS was adjusted to 2.5 m/s (Durrer et al., 1970; Dux-santoy et al., 2012). The electrical propagation in the torso mesh was considered isotropic and specific conductivities were assigned to each organ: (i) myocardium (4.589 mS/cm), (ii) bones (0.200 mS/cm), (iii) liver (0.277 mS/cm), (iv) lungs (0.389 mS/cm), (v) muscle (2.390 mS/cm), and (vi) blood (7.0 mS/cm) based on several experimental studies (Roth, 1992; Bradley et al., 1997; Keller et al., 2010).

### Pathological Model

To simulate LBBB, an electrical block was generated on the left section of the PS before the bifurcation into three sub-branches by imposing null conductivity in two linear elements (see Figure 1D). HF condition was modeled by a reduction of 50% in CV, in accordance with protein connexin43 (Cx43) reduction observed in failing tissue (Coronel et al., 2013). The decrease and lateralization of this protein is associated with reduced longitudinal conduction velocity (Ai and Pogwizd, 2005; Akar et al., 2007; Wang and Hill, 2010).

### Stimulation Protocols

For the present study a 3D anatomical model of the ventricles was generated, which does not include the geometry of the atria. Therefore, the intrinsic activation from the sinoatrial node was simulated by applying an electrical stimulus to the His bundle, either in healthy or HF + LBBB conditions (see Figure 1D). CRT leads were modeled as 0.5 mm^3^ cubes injecting a transmembrane current of 400 μA/μF in amplitude (see Equation 1). Four scenarios of CRT pacing were defined for HF + LBBB conditions with different combinations of atrioventricular delay (AVD) and interventricular delay (VVD) for each lead location configuration (AVD = 100 ms, VVD = 0 ms; AVD = 100 ms, VVD = 30 ms; AVD = 140 ms, VVD = 0 ms; AVD = 140 ms, VVD = 30 ms).

AVD is the time delay between the instant of initial activation of the sinoatrial node (external or intrinsic stimulation) and the instant of time of external CRT stimulation of the ventricles. To set the value of AVD in our simulations, several considerations were taken into account. Firstly, the typical duration of PR interval observed in LBBB patients is 200 ms (Rickard et al., 2017), which is the time that takes the initial atrial stimulation to spread through the atria (100 ms), plus the time delay in the atrioventricular (AV) node (80 ms) (Prinzen et al., 2017), plus the propagation time from the His bundle through Purkinje system to finally reach the first activation site of the ventricles (20 ms approximately). Secondly, our model does not include the atria or the AV node as mentioned before, so that the intrinsic activation was simulated by stimulating His bundle, which is included in our 3D model.

In our simulations, different AVDs could be simulated by changing the stimulation time of the His bundle (coming from the intrinsic activation of the atria). Thus, an AVD of 100 ms was modeled by applying an electrical stimulus to the His bundle of 80 ms after ventricular leads activation. Indeed, when we applied the external CRT ventricular stimulation, 100 ms after initial activation of the sinoatrial node (AVD of 100 ms), this intrinsic activation had reached the atrial side of the AV node (this takes 100 ms) and needed still 80 ms to reach His bundle (delay needed in the AV node). In the case of an AVD of 140 ms, the electrical stimulus in the His bundle was applied 40 ms after ventricular leads activation. Indeed, when we applied the external CRT ventricular stimulation, 140 ms after initial activation of the sinoatrial node, this intrinsic activation had reached the AV node in 100 and 40 ms of delay in the AV have also elapsed, the stimulus needed 40 ms more to reach the His bundle, and this is why we stimulated the His bundle 40 ms after the ventricles. Additionally, VVD was set to 0 ms (stimulation in both ventricles simultaneously) and 30 ms (the RV was stimulated 30 ms after the LV), according to the time ranges used in clinical practice (Brignole et al., 2013; Urbanek et al., 2017).

### Leads Location

**Figure 2:**
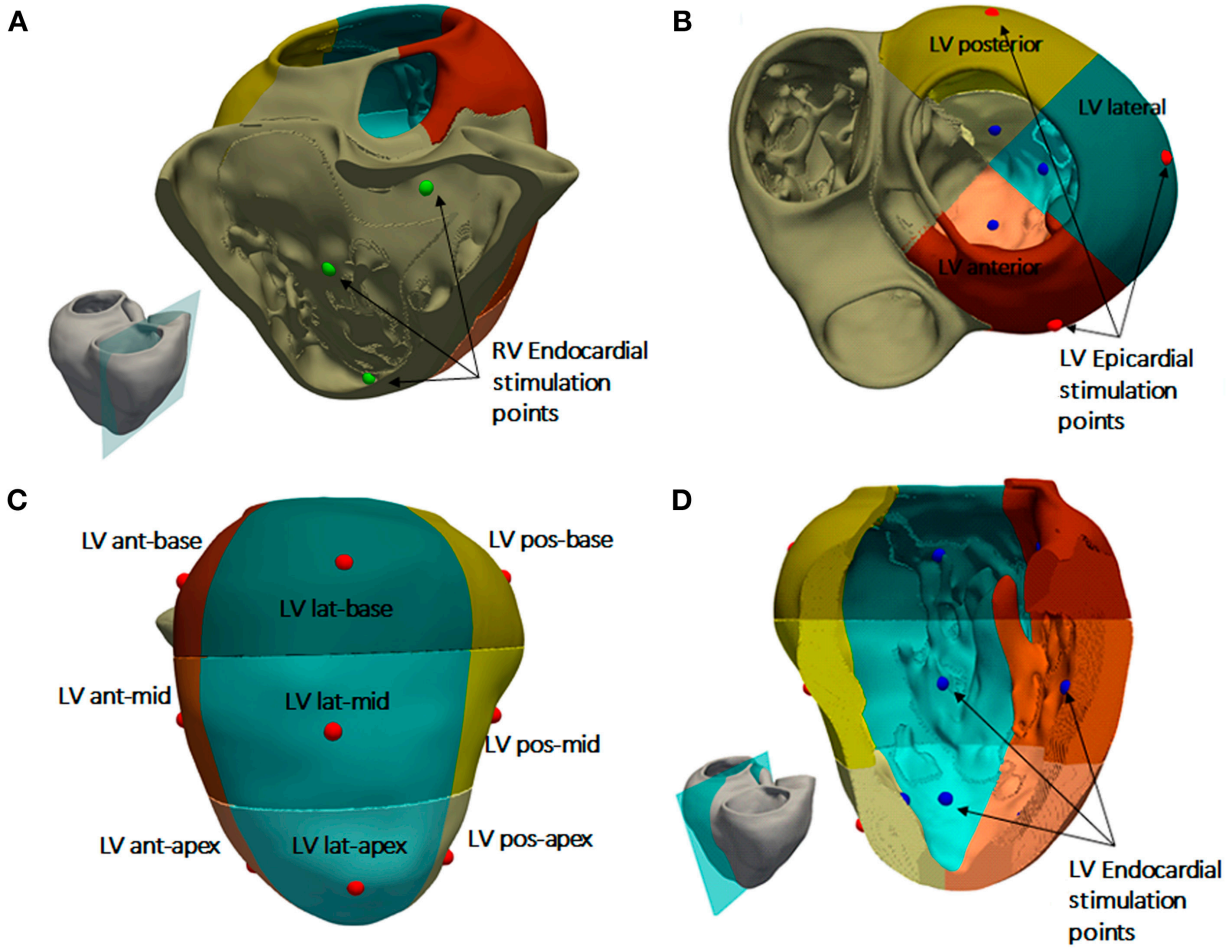
Heart subdivisions and stimulation points for CRT protocol. **(A)** RV septal endocardial stimulation points tested (green). **(B)** Left ventricular (LV) free wall region divided into three regions: posterior (yellow), anterior (brown), and lateral (green). **(C)** Subdivisions of the three LV free wall regions into nine segments. Epicardial stimulation points tested in the middle of each segment (red dots). **(D)** Endocardial stimulation points tested in the LV free wall (blue dots).

The RV septal wall is an alternative location for the RV pacing lead in CRT. In this study, three different locations for the RV pacing lead were tested based on medical protocols and research works (Da Costa et al., 2013; Pastore et al., 2013). The RV septal electrode was placed in the apex (RVapex), middle septal region (RVmid) or upper region near the outflow tract (RVupper) (Figure 2A).

For the LV pacing lead location, the LV free wall was divided into three different regions (Singh et al., 2011; Dou et al., 2012): anterior, lateral, and posterior (Figure 2B). In addition, each region was divided into three segments: apical, mid-cavity, and basal, leading to a set of nine segments for the LV free wall as illustrated in Figure 2C as in Singh et al. (2011). The LV pacing lead was placed in the middle of each segment, both in the epicardial (Figure 2C) and endocardial wall (Figure 2D) to represent and simplify the different possible positions of the electrode within the same region, due to variety of veins configurations observed in CRT patients.

To summarize, we have a total of 54 lead location configurations obtained by combination of the three RV lead locations with 18 LV lead locations (9 epicardial and 9 endocardial) for the application of the CRT protocol.

### QRS Measurements

QRS complex was computed in the precordial leads location on the torso surface for each CRT configuration and QRSd was measured using an algorithm implemented in Matlab software (Mathworks Inc., Natick, MA, USA). This algorithm determines the beginning and end of the QRS complex based on the first and second derivate of the electrocardiographic signal (see Figure S2). The QRS onset was calculated applying a threshold in the first derivate to determine a change in the slope. To estimate the end of QRS complex, additional signal processing was required as baseline was not reached in most CRT configurations. A time interval after the QRS complex was set based on the 95% of the accumulated area under the curve of the second derivate, and the end of the signal. To set the end of the QRS, the lowest value of the first derivate was used within this interval (see Supplementary Material for details). Once the beginning and end of QRS complex were determined for each precordial lead, the QRSd was calculated as the time interval between the onset beginning and the latest end of the QRS among all leads (Surawicz et al., 2009). This is the recommended criterion by the American Heart Association, the American College of Cardiology Foundation, and the Heart Rhythm Society (AHA/ACC/HRS). The total activation time (TAT) of the ventricular mesh was estimated as the time interval between the first and last depolarized node mesh above a threshold of −10 mV.

### Correlation Analysis

Shortest QRSd was the criterion applied to evaluate the optimal location of the LV lead for different positions of the pacing lead in the RV. However, the total activation time (TAT), QRS area (QRSa) and the time to 90% of activated tissue (t_90_) are other important parameters that have been used to evaluate CRT response. For this reason, three linear correlations between these parameters were performed using Pearson correlation method. Values of *p* < 0.05 were considered statistically significant. Values for the analysis are shown in Tables S1–S5.

## Results

### Model Validation

**Figure 3:**
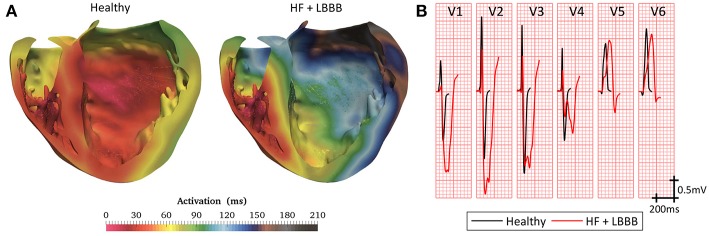
Model validation. **(A)** Cross section of biventricular model showing color coded local activation maps of a healthy (left) and pathological heartbeat (right). **(B)** Precordial leads signals recorded on torso surface.

Simulated ventricular activation maps for non-pathological and HF conditions with LBBB (HF + LBBB) are shown in Figure 3A. In healthy conditions, the electrical impulse traveled from the bundle of His to the first activation point in the LV endocardium in approximately 20 ms. RV activation started 10 ms after the onset of LV activation (Durrer et al., 1970). The computed time until all the ventricular tissue was depolarized (total activation time or TAT) was approximately 103 ms, in accordance with human data (Boukens et al., 2014). The outflow tract and the posterobasal area were the last activated regions in the RV, while the latest areas depolarized in the LV were the anterior mid and basal regions.

Under HF + LBBB conditions, activation began in the RV endocardium and reached the LV endocardium in the apical septal region after 46 ms from the onset of the RV depolarization. This is in agreement with the data recorded experimentally by Auricchio et al. (2004). The last activated region in the LV was the lateral wall in accordance with the study of Mafi Rad et al. (2014). Additionally, the TAT was increased in 104% compared to a healthy heart.

Figure 3B shows the computed QRS complexes in the precordial leads for simulations in a healthy heart and under HF + LBBB conditions. For non-pathological conditions, QRS duration (QRSd) was 93 ms, while in HF + LBBB QRSd was increased to 190 ms. Both values are within experimental ranges (Spragg et al., 2010; Tian et al., 2017). Additionally, QRS complexes in HF + LBBB simulations present an rS pattern (small R wave followed by a bigger S wave) (Sweeney et al., 2014) in leads V1 and V2 and a mid-QRS notching in several leads. These observations are in agreement with the criteria proposed by Strauss et al. (2011) to define complete LBBB.

### QRS Duration During CRT

A total of 54 electrode placement configurations with four different delays (two AVD and two VVD configurations) settings were tested for the CRT simulations. QRSd values are shown in Table S1.

**Figure 4:**
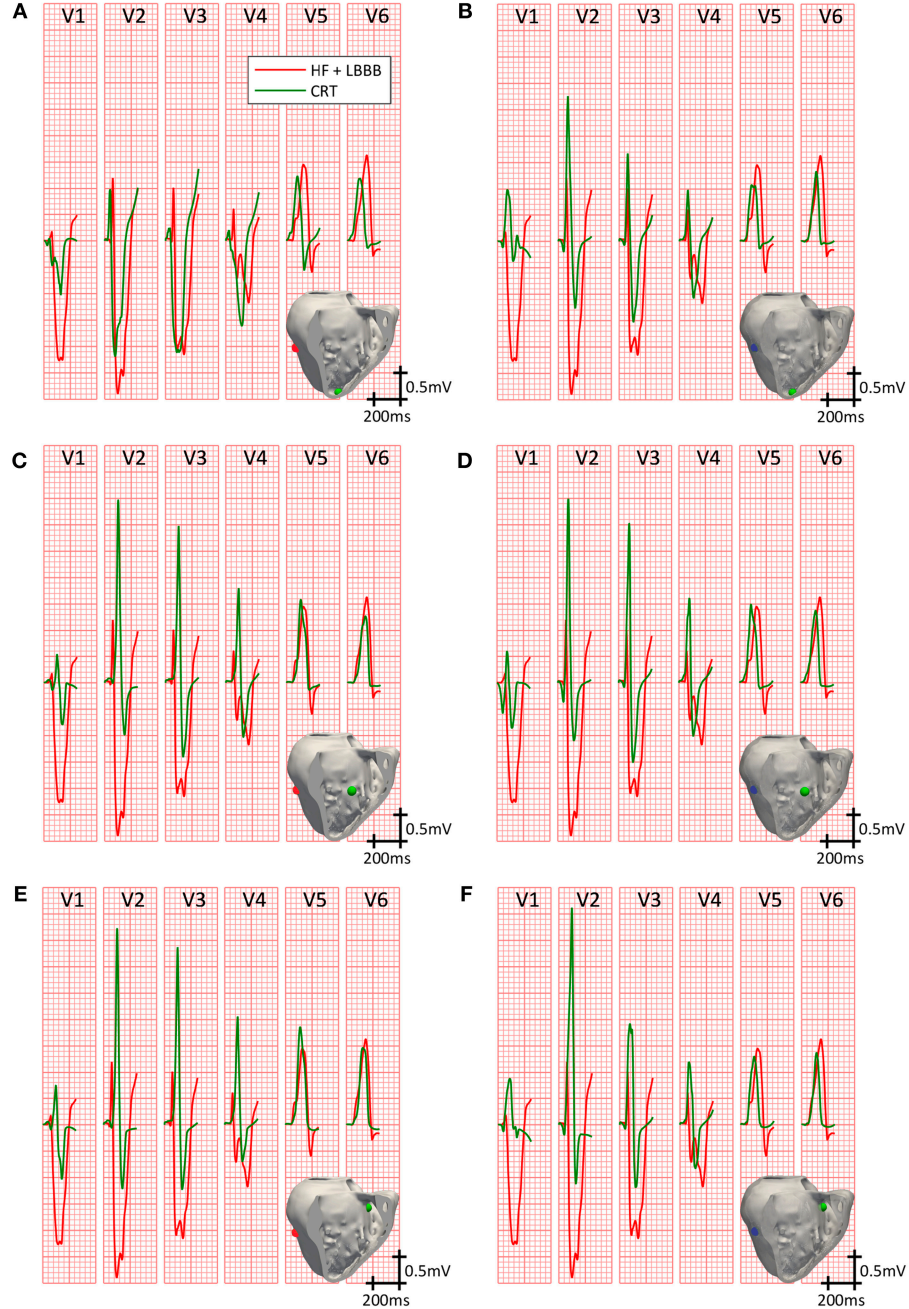
Precordial leads signals on CRT. QRS complexes in the precordial leads under HF + LBBB conditions, before (red trace) and after (green trace) the application of the best CRT configurations (shorter QRSd). Three different locations for the RV pacing lead were tested: RV apex with epicardial **(A)** and endocardial **(B)** LV lead stimulation; RV mid septum with epicardial **(C)** and endocardial **(D)** LV lead stimulation; and RV upper septum with epicardial **(E)** and endocardial **(F)** LV lead stimulation. Stimulation points are shown in light green inside the insets for the RV lead, and in blue and red for the LV endocardial and epicardial lead, respectively.

Figure 4 compares the simulated QRS complexes in a scenario with HF + LBBB before (red traces) and after (green traces) the application of the CRT protocol. The optimal configurations in terms of shortest QRSd for the RV lead placement tested (apex, mid septum, and upper septum) are shown in the different rows. Epicardial vs. endocardial LV lead stimulation for those configurations are shown in columns.

Firstly, we analyzed the optimal lead placement. The shortest QRSd among all configurations tested was obtained when the RV lead was placed in the upper septum near the outflow track (third row). Furthermore, for all RV lead placement the optimal location of the LV lead, both in the epicardium and endocardium, was the LV mid posterior wall.

Secondly, we analyzed the effect of the delay between pacing leads and intrinsic activation in a fixed location. The best configurations for the RV lead placed in the apex are depicted in the first row. QRSd was reduced from 172 ms (Figure 4A) to 157 ms (Figure 4B), but bigger reductions were obtained for different intrinsic and pacing delays (AVD = 140 ms, VVD = 0 ms; AVD = 100 ms, VVD = 30 ms, respectively).

When the RV lead was located in the middle of the septum (second row), the QRSd was reduced from 161 ms (Figure 4C) to 146 ms (Figure 4D) for optimal configurations. In this case, these results were obtained for different pacing delays between leads but the same AVD (AVD = 140 ms and VVD = 30 ms vs. AVD = 140 ms and VVD = 0 ms, respectively).

If the RV lead was placed in the upper septum, the QRSd was reduced from 149 ms (Figure 4E) to 143 ms (Figure 4F). However, in this case both configurations were achieved with the same intrinsic and biventricular delay (AVD = 140 ms, VVD = 30 ms).

Finally, the influence of LV epicardial vs. endocardial pacing was assessed. QRSd was decreased in all cases after CRT application, but the reduction was greater for LV leads placed in the endocardium (column 2) compared to epicardium (column 1).

Summarizing, the optimal location in terms of shortest QRSd was obtained when the RV lead was placed in the upper septum and the LV lead was located in the mid posterior wall region. Once the optimal lead location was selected for both RV and LV leads, the shortest QRSd was measured for different intrinsic and biventricular delays, without highlighting a particular optimal setting. Finally, the shortest QRSd was obtained in all configurations when the LV lead was placed in the endocardium compared with those in the epicardium.

### Ventricular Activation Time During CRT

**Figure 5:**
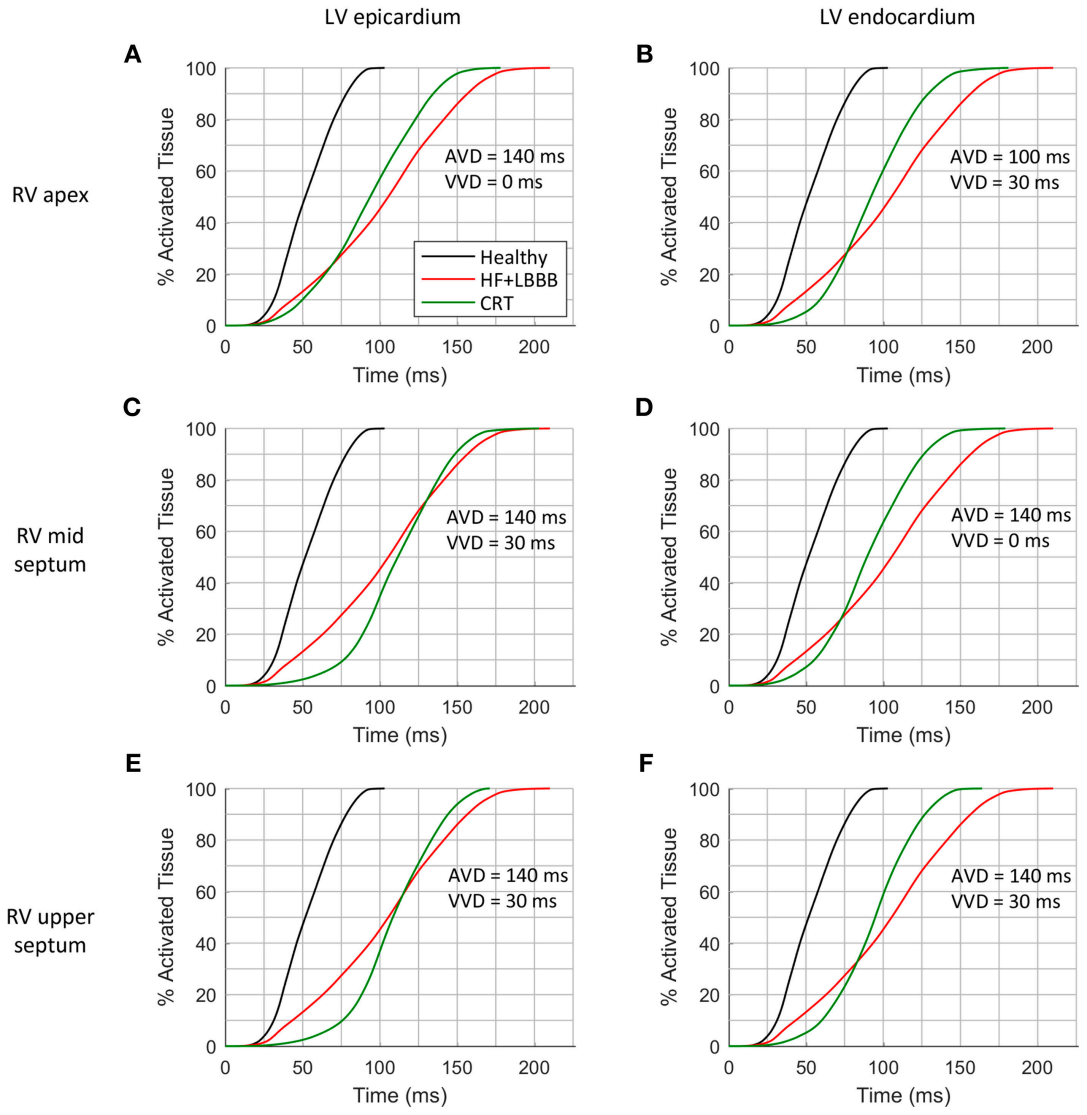
Cumulative frequency histograms of the normalized percentage of activated tissue. The curves correspond to healthy (black), HF + LBBB (red) and CRT (green) scenarios. The best CRT configurations (shortest QRSd) for the three locations of the RV lead were tested: RV apex with epicardial **(A)** and endocardial **(B)** LV lead stimulation; RV mid septum with epicardial **(C)** and endocardial **(D)** LV lead stimulation; and RV upper septum with epicardial **(E)** and endocardial **(F)** LV lead stimulation.

Another helpful parameter to assess CRT outcome is the total activation time (TAT) of the ventricles. This parameter is not directly accessible in clinical practice during CRT procedures, but simulations can provide additional information to achieve the ideal configuration. Ideally, within normal physiological ranges, the shorter the QRS the shorter TAT, leading to an increase in ventricular synchrony. In Figure 5, the percentage of activated ventricular tissue is shown as a function of time for the healthy heart, under HF + LBBB conditions, and for the optimal CRT configurations (as a function of RV location), which are shown in Figure 4. Under HF + LBBB conditions (red trace), the electrical impulse spreads throughout the ventricles much slower (gradual slope) than in the healthy heart (black trace) or in CRT (green trace) configurations, completing ventricular activation after 210 ms. For CRT simulations, the rate of activated tissue was initially low, but increased rapidly to reach rates similar to those observed in healthy cases. This was especially noticeable when the LV lead was located in the epicardium (first column) and the RV lead was located in the mid and upper septum (Figures 4C,E, respectively). These results can be explained because of several factors. Firstly, the configuration of the PS and the PMJ distribution strongly affects the initial spread of the wavefront. Given the PS RV morphology, i.e., two main branches, one descending to the apex and another growing around the moderator band (Figure 1D), when the RV lead was located in the apex, the electrical stimulus entered fast in the PS (around 5 ms) and propagated to remote areas faster than through the myocardium (see Video 1, CRT). However, it took around 40 ms to retrogradely enter in the PS when the RV lead was located in the mid septal region, and around 90 ms when the RV lead was in the upper septum. For this reason, it took 75 ms to activate initially only 10% of the myocardium. Secondly, stimulation in the epicardial layer took longer to reach PMJ locations. Thirdly, the stimulation delay between both ventricles (VVD) also affected the initial slope of cardiac activation. Nevertheless, after 70 ms for the endocardial configurations (second column) and 125 ms for the epicardial ones the percentage of activated tissue during CRT application was higher than the percentage of HF + LBBB conditions. Moreover, during the final phase of ventricular activation, the rising rate was considerably reduced. Indeed, the electrical impulse took between 26 and 54 ms (15% to 26% of the TAT) to activate the last 10% of the ventricular tissue.

Finally, after applying the CRT protocol, the TAT was decreased by 15, 14, 12, 15, 19, 22%, with respect to HF + LBBB conditions, as shown in Figures 5A–F, respectively. The locations of the pacing leads for the shorter QRS complexes coincided with the locations of the electrodes for the shorter TAT. However, when VVD and AVD were modified, the shortest QRS did not match the shortest TAT, which means that QRSd and TAT are not totally correlated. In addition, the difficulty in QRS measurement at the beginning and end of the signals has to be considered.

**Figure 6:**
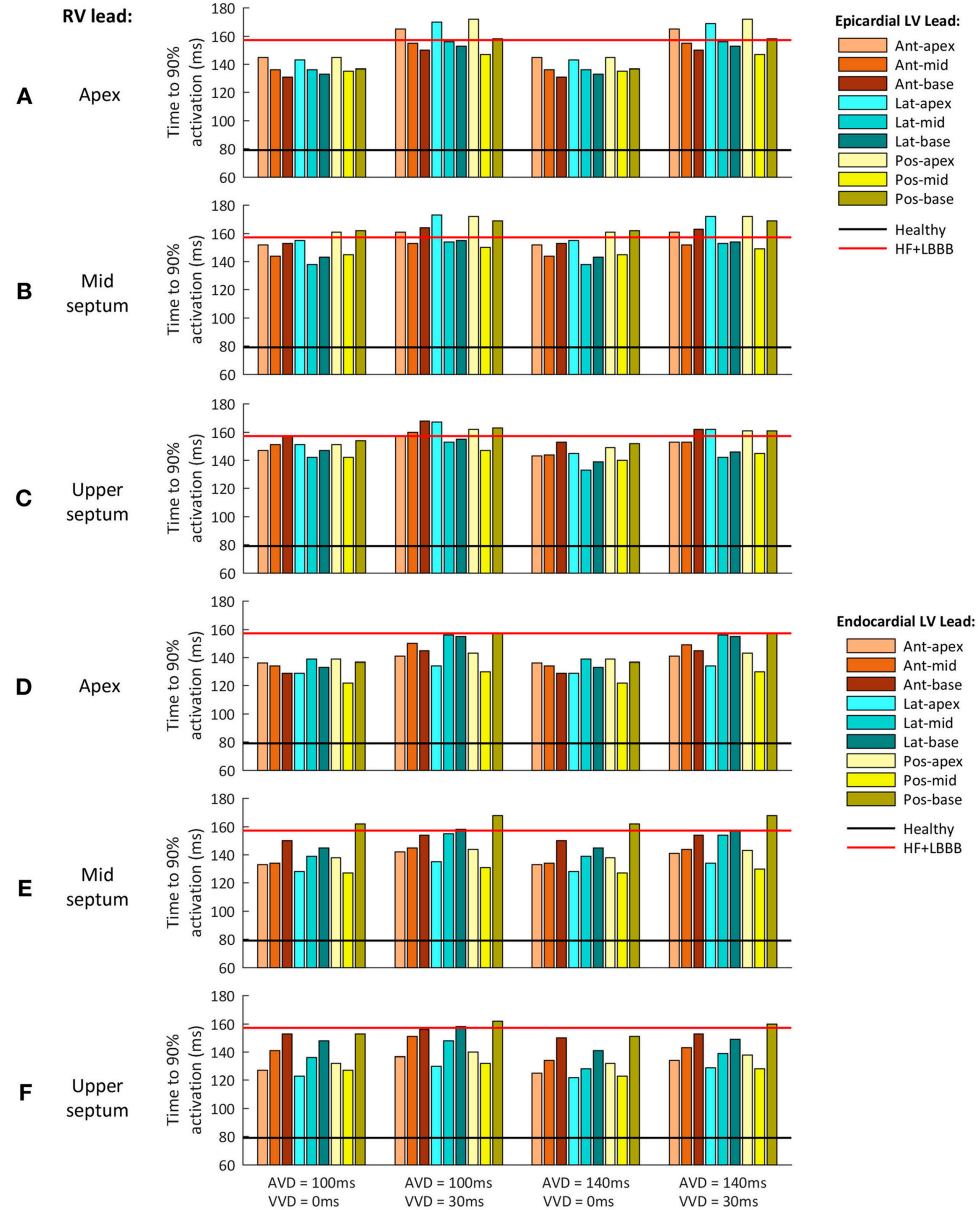
Time to 90% of ventricular activation for the different CRT configuration delays assessed. **(A–C)** Epicardial LV lead stimulation for the three RV lead location tested: **(A)** RV apex, **(B)** RV mid septum and **(C)** RV upper septum. **(D–F)** Endocardial LV lead stimulation for the three RV lead location tested: **(D)** RV apex, **(E)** RV mid septum and **(F)** RV upper septum. The three LV regions (anterior, lateral and posterior walls) are shown in different color brightness (red, blue and yellow). The values for healthy and HF + LBBB configurations are depicted in black and red lines respectively.

In clinical practice, a shorter QRSd is one of the standard criteria used to evaluate CRT response. However, both non-responder and responder patients show a reduction in QRSd after CRT application (Molhoek et al., 2004; Elhakam Elzoghby et al., 2017). Therefore, an additional indicator would be useful for a better perception of CRT benefit. As shown in Figure 5, TAT could be strongly modified by the initial rate of activation, as well as by the last activation interval. To avoid this, we analyzed the time elapsed to 90% of ventricular activation (t_90_), (Figure 6). This parameter allows us to determine which configuration leads to a faster activation of most of the ventricular tissue, thus decreasing electrical dyssynchrony.

Figure 6 shows t_90_ values for a configuration with the RV lead placed in the apex, mid septum, and upper septum, and the LV lead located in the epicardium (Figures 6A–C), and the same RV configurations with the LV located in the endocardium (Figures 6D–F). The different delays applied between the His Bundle and CRT leads (AVD) and between the RV and LV leads (VVD) are shown in columns.

The optimal location of the LV pacing lead, both in the epicardium and endocardium, changed during CRT application for each of the pacing lead locations in the RV. However, the optimal AVD and VVD were the same in all cases, 140 and 0 ms (third column), respectively.

On the one hand, when AVD was modified (column 1 vs. column 3 and column 2 vs. column 4) similar results were obtained, except when the RV lead was located in the upper septum area. The electrical propagation of the intrinsic stimulus contributed to decrease t_90_ (7% reduction) for an AVD of 140 ms. On the other hand, when VVD was increased (column 1 vs. column 2 and column 3 vs. column 4) t_90_ increased up to 19% for all the RV lead locations.

When the RV lead was located in the apex, the optimal location of the LV lead in the epicardium was the LV anterior wall at basal level (Figure 6A). For the same RV lead location, the optimal LV lead location in the endocardium was the LV posterior wall at mid-cavity level (Figure 6D). Changing the RV lead location to mid septum, the optimal LV lead location in the epicardium was the LV mid lateral wall, while the optimal LV lead location in the endocardium was the LV mid posterior wall (Figures 6B,E, respectively).

Finally, for the RV lead location in the upper septum, the optimal placement of the LV pacing lead in the epicardium was in the LV mid lateral wall, while the optimal placement of the LV lead in the endocardium was the apex of the LV lateral wall (Figures 6C,F, respectively). Table 1 summarizes the optimal placement of the LV lead for a faster activation of 90% of the ventricular tissue. The optimal locations calculated are not in agreement with the optimal RV lead location determined based on a shorter QRSd in most cases. This result suggests the hypothesis that the shortest QRSd does not necessarily imply the fastest ventricular activation of 90% of the ventricular muscle.

**Table 1:** Optimal placement of the LV lead on CRT.

**Criterion**	**RV lead**	**LV lead**	**AVD (ms)**	**VVD (ms)**
**LV EPICARDIAL STIMULATION**				
Shortest QRS duration	Apex	Posterior - mid	140	0
	Mid septum	Posterior - mid	140	30
	Upper septum	Posterior - mid	140	30
Shortest TAT	Apex	Posterior - mid	140	0
	Mid septum	Posterior - mid	140	0
	Upper septum	Posterior - mid	140	0
Faster activation of 90% of the ventricular tissue	Apex	Anterior - base	140	0
	Mid septum	Lateral - mid	140	0
	Upper septum	Lateral - mid	140	0
**LV ENDOCARDIAL STIMULATION**				
Shortest QRS duration	Apex	Posterior - mid	100	30
	Mid septum	Posterior - mid	140	0
	Upper septum	Posterior - mid	140	30
Shortest TAT	Apex	Posterior - mid	140	0
	Mid septum	Posterior - mid	140	0
	Upper septum	Posterior - mid	140	0
Faster activation of 90% of the ventricular tissue	Apex	Posterior - mid	140	0
	Mid septum	Posterior - mid	140	0
	Upper septum	Lateral - apex	140	0

*TAT, total activation time; AVD, atrioventricular delay; VVD, interventricular delay*.

### Correlation Between Ventricular Activation and QRS

**Figure 7:**
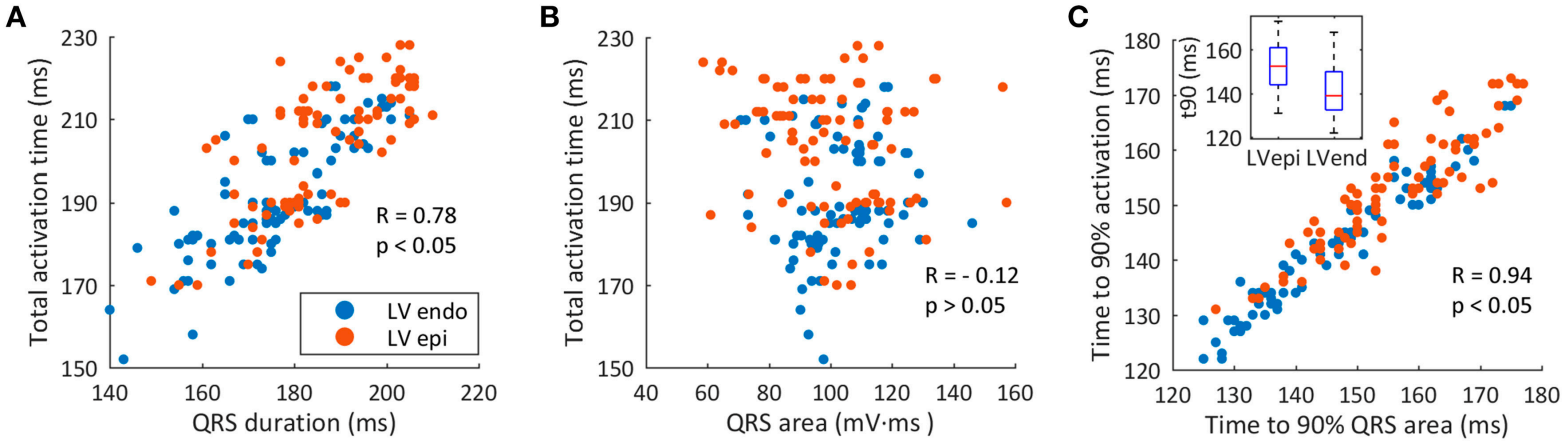
Correlation between ventricular activation and QRS. **(A)** Correlation between QRS duration and TAT (red circles show LV epicardial leads, blue circles show LV endocardial leads). **(B)** Correlation between the QRS area and TAT. **(C)** Correlation between t_90_QRSa and t_90_.

To better highlight the relationship between QRSd and TAT, a correlation analysis was carried out (Figure 7A). Results showed an elliptical distribution of data with a moderate positive linear relationship, statistically significant (*R* = 0.78 and *p* < 0.05). This moderate correlation could justify the difference between the optimal AVD and VVD values for the simulations with a shortest QRSd and with a shortest TAT.

A similar correlation analysis was made between area of the QRS (QRSa) and TAT (Figure 7B). We first calculated the QRSa for the average signal of the six precordial leads, between the beginning and end values determined during the measurement of the QRSd. The results of the correlation show a scattering distribution of data with a statistically non-significant *p*-value (*R* = −0.12 and *p* = 0.076). Thus, a linear relationship between QRSa and TAT was not observed in this study.

Finally, when correlating the curves of percentage of activated tissue and percentage of QRS area as a function of time, a direct relationship between both variables was observed. Figure 7C shows the correlation between time to 90% of QRSa (t_90_QRSa) and time to 90% of the ventricular activation (t_90_) for each CRT simulations. A significant correlation with a high linear dependence was observed (*R* = 0.94 and *P* < 0.05). Simulations with shorter t_90_QRSa correspond to the simulations with shorter t_90_. Therefore, a new biomarker based on time up to 90% of the QRS area can be used as an indicator of electrical synchrony.

## Discussion

In this study, biophysical 3D multiscale simulations were conducted to assess alternative locations of the RV lead for a better CRT response in LBBB HF patients. The major findings of this study can be summarized as follows: (i) the optimal leads location based on shortest QRS criterion was the RV upper septum and the LV mid posterior region minimizing also TAT; (ii) for the optimal lead location, the delay configuration leading to the shortest QRSd was AVD = 140 ms, VVD = 30 ms. However, the AVD and VVD setting leading to the shortest TAT was different, suggesting that minimizing QRSd is a good criterion to select leads location but not to select the pacing delay; iii) the time to 90% of the QRS area (t_90_QRSa) was a good predictor of the instant at which 90% of the ventricular tissue had been activated (t_90_). This indicator could be used in clinical trials to complement QRSd criterion to select the optimal delay of the pacing leads to obtain a faster ventricular activation of most of the ventricular muscle.

### Optimal Lead Location

The location of the optimal pacing site varies significantly between patients, so that a strategy of individualized LV lead placement is required to maximize the benefit of CRT (Derval et al., 2010; Spragg et al., 2010). The apex for permanent LV pacing should be avoided, as this location has been associated with poor outcomes in studies such as MADIT-CRT (Yoshikawa et al., 2010; Singh et al., 2011). The experimental study PATH-CHF I suggested that the mid lateral left ventricular site for the LV lead may show greater acute benefit in patients with LBBB (Auricchio et al., 1999). In general, a lateral or posterior vein is the desired location for achieving optimal hemodynamic support as this is usually the site of most delayed activation of the left ventricular wall in patients with LBBB (Stellbrink et al., 2000; Singh et al., 2006).

Our simulation results suggested the upper area of the RV septum as the optimal position for the RV lead, in agreement with some experimental (Muto et al., 2007; Flevari et al., 2009; Cano et al., 2010; Da Costa et al., 2013) and simulation (Miri et al., 2009a) studies. The study of Leclerq and coworkers (Leclercq et al., 2016) demonstrates that septal and apical RV pacing in CRT have a similar clinical outcome and similar LV reverse remodeling after 6 months of therapy. However, other studies (Victor et al., 2006) reported the shortest QRSd for RV septum pacing but not a better CRT response (similar LVEF at 6 months). This highlights the need of additional indicators to determine the optimal placement of the pacing leads.

In the present simulation study the most delayed activation area was located in the anterior basal LV region in the HF + LBBB configuration under intrinsic activation. We also assessed the latest activated area of the LV, when only RV stimulation was applied. If the RV lead was placed in the apex, the anterior basal LV area was activated the latest. However, the LV lateral wall was the latest activated area when the RV lead was located either in the middle or upper septal regions (see Figure S3).

The study of Zanon et al. (2014) determined that the LV lead location in the latest activated site was predictive of the maximum increase in contractility (LV *dP/dt*_max_). On the other hand, in the recent study of Sipal and coworkers (Sipal et al., 2018), comparing the clinical benefits of LV lead implantation guided by the shortest BiV-paced QRSd using surface ECG and with the standard unguided CRT, there was a significantly higher rate (85 vs. 50%, *p* = 0.02) of response (>15% reduction in LV end-systolic volume) to CRT as well as a shorter QRSd (*p* < 0.001) and a greater QRS shortening for the surface ECG guided group. Furthermore, the optimal site for LV lead placement was the posterior and posterolateral region, in agreement with our simulations. For all RV lead locations tested in our study, when the LV lead was placed in the latest activated area of the LV, none of those configurations led to the shortest QRSd.

In our study, we also showed that when pacing in the latest electrically activated area of the LV, that area did not provide the shortest TAT. Similar results were observed in the simulation study by Pluijmert et al. (2016). In that work, the authors found that the LV pacing region that provided the maximum acute hemodynamic response, located near the latest activated area, did not lead to the largest reduction of TAT during biventricular stimulation. Even stimulating regions leading to the largest reduction of TAT showed poor increase of hemodynamic response. However, other studies have found a positive correlation between acute hemodynamic response and TAT reduction (Crozier et al., 2016). The optimal method to place the LV pacing lead is thus object of controversy: while several studies support that pacing in the latest activated area leads to better hemodynamic response, others consider the criterion of maximal reduction in QRSd as the best choice.

### Optimal Delay Between Pacing Leads

Optimization of AVD and VVD is crucial during CRT application. A longer inter-lead electrical delay was associated with more pronounced LV reverse remodeling in CRT patients with a presumed optimal LV lead position concordant or adjacent to the latest mechanically activated non-scarred segment (Sommer et al., 2018).

In clinical practice this value should be specifically set for each patient, although optimization is rarely performed in the real practice. The largest trials studying CRT used various methods to optimize these intervals, most frequently based on echocardiography and intracardiac electrogram interval measurement, but unequivocal proof of the benefit brought by optimization is still lacking (Abraham et al., 2010; Krum et al., 2012; Brugada et al., 2014). Echocardiography presents inherent variability of results and is highly operator dependent (Gras et al., 2009). Optimization based on intracardiac electrogram intervals has not proved yet to be of clear benefit above arbitrary AV interval (Ulč and Vančura, 2013). Multisite pacing has shown favorable results, although it is technically complex (Cazeau et al., 2001). A less time-consuming and easier optimization method might enable a more systematic optimization of the AVD and VVD at routine follow-up visits in all recipients of CRT systems.

The morphology of the PS clearly determined in our study the influence of AVD. When the RV lead was placed in the apex, the intrinsic activation of the His bundle found the majority of the Purkinje network already depolarized via retrograde conduction. However, if the RV lead was placed in the middle septum or closer to the outflow tract, further from any possible entrance to the cardiac conduction system, the intrinsic depolarization wavefront spread faster to the myocardium than the wavefront generated through the CRT leads, leading to a reduction in TAT. Some experimental studies (Prinzen and Peschar, 2002) support the idea that PS may not allow retrograde conduction in LBBB patients due to structural damage, or if allowed, reduced conduction velocity would be observed in LV PS sections, neglecting the influence of PS. Whether the rest of the LV branches are able to conduct retrogradely (Huang et al., 2017) or other areas of the Purkinje network deteriorate, as HF evolves, remains unknown. Experimental studies have measured a strong reduction in septal conduction velocity during LBBB when HF was advanced compare to acute LBBB (Strik et al., 2013). In that case, the simulation results of this study should be considered with caution. Although new methodologies are arising to better describe the PS (Lee et al., 2014; Barber et al., 2018). The lack of technology to characterize the PS in a patient specific manner, limits the optimal configuration for CRT.

Traditional CRT pacing mode does not promote ventricular activation through conduction system from the sinoatrial node. The lack of enough information on the chronic effects of the fusion leads (intrinsic stimulation combined with external pacing) and this method is avoided, setting the shortest AVD based on echocardiography (Barold et al., 2008). In our study, a fusion between the intrinsic activation and biventricular (BiV) pacing for the optimal CRT configuration (pacing lead location and delays) was assessed. Several experimental works support this procedure (Van Gelder et al., 2005; Vatasescu et al., 2009; Arbelo et al., 2014; Guo et al., 2015). Guo et al. determined that congestive heart failure patients with BiV pacing + intrinsic activation presented improvement in cardiac function and quality of life (Guo et al., 2015). Meanwhile, Vatasescu and coworkers observed that BiV pacing fused with intrinsic activation might increase the rate of structural responders (Vatasescu et al., 2009).

Biophysical models of the heart have been used to optimize AVD, VVD and lead location during CRT simulation (Miri et al., 2008, 2009a,b; Pluijmert et al., 2016; Lee et al., 2017). QRSd, estimated as the difference between the time of the first and last activated cardiac cell (or TAT), have been used as one optimization criterion by Miri and coworkers. In our study, the optimal LV lead location based on the shortest QRSd (calculated in the ECG signal) was similar to the region with shortest TAT (see Table 1). However, VVD value that produced the shortest QRSd did not match with the VVD that produced the shortest TAT, which means that QRSd and TAT are not totally correlated. A simulation study by Potse et al. (2012) support this result. The authors observed that biventricular pacing did not change QRS duration but reduced total ventricular activation time when the LV stimulation was applied in one point of the LV free wall.

### Indicators to Evaluate CRT Outcome

The Echocardiography Guided Cardiac Resynchronization Therapy (EchoCRT) study further reinforced the importance of QRSd over mechanical dyssynchrony as the most important indicator for CRT responses (Ruschitzka et al., 2013). Other studies have proposed indexes based on QRS measurements. Van Gelder et al. (2009) showed a relation between the Q-LV interval (the interval from Q wave to intrinsic deflection on the LV EGM) and the acute hemodynamic effect on optimized biventricular stimulation. A longer Q-LV interval predicted a greater increase in LV pressure rise (LVdP/dt_max_) and vice versa. Normalizing the QLV by QRS duration, termed LV lead electrical delay (LVLED), was also shown to correlate with Doppler-derived dP/dt values. LVLED greater than or equal to 50 % was associated with significantly greater reductions in all-cause death or HF hospitalization at 12 months of follow-up in patients with non-ischemic cardiomyopathy (Singh et al., 2006).

Our simulations show that the difference in QRSd was significant when the LV was paced in different sites and for a fixed placement of RV. However, these differences in QRSd were decreased when adjusting the delay between leads in a fixed location for both leads (see Table S1). Thus, the shortest QRSd predicted precisely the region in the LV subdomains that produced the shortest TAT for the three locations of the RV lead tested, leading to an increase in ventricular synchrony. However, this index could not determine the pacing delay configuration between leads that allows to obtain the shortest TAT. There is no consensus on how QRS should be accurately measured, and therefore small differences are expected between methods (De Pooter et al., 2016). In our study, the optimal QRSd obtained after CRT application supposed a 20% reduction of the QRSd. This result is in agreement with the study of Elhakam Elzoghby et al. (2017), where 180 patients under heart failure conditions and LBBB were studied, and similar reductions were obtained. Other studies obtained lower QRS reduction values, namely 17 and 12%, for CRT responders in Molhoek et al. (2004) and Pitzalis et al. (2002) studies, respectively.

The assessment of interventricular dyssynchrony was done analyzing the TAT. Our results showed that a shorter duration of the QRS complex is moderately correlated with a shorter TAT (Figure 7A). The narrowest QRS complex predicted the optimal location of the stimulation leads but not the optimal value of the VVD. In this way, the t_90_ index selected correctly the best delay configuration to provide the fastest activation of the majority of the heart. In several configurations, TAT value was exactly the same (see Table S2), but t_90_ discerned the shortest order of activation. Thus, the shortest QRSd predicted the location for the optimal leads placement, but t_90_ predicted the best pacing delay with the shortest TAT. We hypothesized that setting the pacing delay properly with this new index could improve CRT non-responders rate.

Other simulation studies have assessed the evolution of TAT during CRT (Romero et al., 2010; Potse et al., 2012) focused on the assessment of the LV intraventricular delay. The recent study of Tomassoni (2016) showed how CRT response assessment is highly variable depending on the criteria used to define the response. QRS width has been shown to correlate well with interventricular dyssynchrony but unfortunately this has poor accuracy for detecting intraventricular dyssynchrony. As a result, it is estimated that only 70% of patients with LBBB have echocardiographic evidence of mechanical dyssynchrony (Bleeker et al., 2004). The role of mechanical dyssynchrony for improving patient selection for CRT remains controversial. The multicenter, nonrandomized Predictors of Response to CRT (PROSPECT) study evaluated the ability of 12 echocardiographic indices of dyssynchrony to predict CRT responses at 6 months (Chung et al., 2008). These indices provided only modest sensitivity and specificity, and researchers reported large variability in quantification of dyssynchrony. Mechanical dyssynchrony has also been used to select CRT candidates with a narrow QRS duration ≤ 120 ms, with limited success in randomized multicenter studies. In this line, mechanical response generated by electrical excitation (excitation-contraction coupling) could be different depending on the heart region (Gurev et al., 2010). Multiple simulation studies have addressed CRT from different perspectives. The recent work of Lee et al. (2018) organized and summarized the state of the art of computational modeling for CRT.

To our knowledge, and given the benefits of using a model where all variables are accessible, our study is the first to systematically explore the correlation between the activated portion of tissue (less accessible in clinical practice) and the QRS complex in the torso surface. Thus, we found that an index based on time to 90% of the QRS area (t_90_QRSa) is a good predictor of the instant at which 90% of the ventricular tissue has been activated (t_90_). This indicator could be used in clinical trials to complement QRSd measurements in defining the optimal location and delay of the pacing leads to produce faster ventricular activation of most of the ventricular muscle.

### Epicardial vs. Endocardial Pacing

Although LV epicardial stimulation decreased QRS width in most cases, a greater reduction was observed for endocardial pacing. The study conducted by Spragg et al. (2010) showed that CRT administered at the optimal site of the LV endocardium was more effective than stimulation through an electrode in the coronary sinus. There is evidence to suggest that endocardial stimulation yields to more natural transmural activation patterns and a better response for CRT patients (Prinzen et al., 2009; Bordachar et al., 2010; Behar et al., 2016). In this line, new devices that allow endocardial pacing and single lead stimulation (Sperzel et al., 2015) coordinated with intrinsic activation will provide new possibilities.

The better results obtained with endocardial pacing are strongly influenced by PS. As PMJs are located in the endocardial surface, the wavefront generated for the LV lead gets into the Purkinje conduction system retrogradely and spreads faster to other inactivated areas (see Video 2, RETROGRADE). Thus, knowing the distribution and location of PMJ, as well as the conduction system morphology is a determinant factor for CRT improvement.

### Limitations

CRT was analyzed only from an electrical point of view in our study. Mechanical behavior based on echocardiography is a common alternative to assess hemodynamic response, although this method is time-consuming and the optimal measurements remain unclear. Simulation studies including the mechanical behavior would be certainly enlightening.

In this study, a particular heart geometry and PS were assessed. The inclusion or not of the moderator band (which may be very patient-specific) may affect QRSd and TAT measurements, especially when pacing on the RV upper septal area. Although our results have been compared to other related studies, the specific findings observed in this study should be carefully validated against clinical studies and complemented with a set of computational models of different patients. In addition, two isolated stimuli were employed to assess CRT efficiency. The development of strategies that allow multi-site pacing should be taken into account in future studies. Additionally, the incorporation of levels of HF in different ventricular areas could modify simulation results.

## Conclusion

In conclusion, our study showed that the optimal location for the RV lead in CRT site as an alternative to RV apex, is the upper septum close to the outflow tract, based on the shortest QRSd criterion. Furthermore, LV endocardial pacing leads improve CRT outcome, with respect to epicardial stimulation, and areas with a higher density of PMJ are suggested for better CRT response.

CRT optimization based only on the shortest QRSd criterion may not be totally effective to reach the maximum TAT reduction, or to optimize biventricular pacing delay. However, a biomarker based on minimizing the value of t_90_ (time elapsed to 90% of ventricular activation) could be used to determine the optimal VVD value. The time to reach the 90% of the QRS area (t_90_QRSa) is related to the time at which 90% of the ventricles are already activated (t_90_). Thus, t_90_QRSa is suggested as an additional index to assess CRT effectiveness to improve biventricular synchrony.

## Author Contributions

EFC, EC, JA, JF, and BT contributed to the conception and design of study. AL-P created the anatomical model and Purkinje system was developed by RS and JG. Computational simulations were performed by EFC and JG. The analysis/interpretation of results was performed by EFC, JG, RS, and BT. All authors contributed to draft the manuscript and all authors revised and approved the submitted version.

### Conflict of Interest Statement

The authors declare that the research was conducted in the absence of any commercial or financial relationships that could be construed as a potential conflict of interest.

## Abbreviations

AV: atrioventricular
AVD: atrioventricular delay
BiV: biventricular
CRT: cardiac resynchronization therapy
CV: conduction velocity
dV/dt_max_: maximum upstroke velocity
FEM: finite element method
HF: heart failure
LBBB: left bundle branch block
LV: left ventricle
LV dP/dt_max_: LV pressure rise
LVEF: LV ejection fraction
LVLED: LV lead electrical delay
PMJ: Purkinje myocardial junction
PS: Purkinje system
QRSa: QRS area
QRSd: QRS duration
RV: right ventricle
TAT: total ventricular activation time
t_90_: time to 90% of the ventricular activation
t_90_QRSa: time to 90% of the QRS area
VVD: interventricular delay.
