# Supplementary material for: Optimization of Lead Placement in the Right Ventricle During Cardiac Resynchronization Therapy. A Simulation Study
Source: Front Physiol. 2019 Feb 11;10:74. doi: 10.3389/fphys.2019.00074 (PMC6378298; doi:10.3389/fphys.2019.00074)
Supplement: Supplementary file 3 [file Data_Sheet_1.docx]

Supplementary Material

Optimization of Lead Placement in the Right Ventricle During Cardiac Resynchronization Therapy. A Simulation Study

Edison F. Carpio, Juan F. Gomez, Rafael Sebastian, Alejandro Lopez-Perez, Eduardo Castellanos, Jesus Almendral, Jose M. Ferrero, Beatriz Trenor^*^

*** Correspondence:** Beatriz Trenor: [btrenor@eln.upv.es](mailto:btrenor@eln.upv.es)

# Supplementary Data

Original O’Hara et al. formulation (O’hara et al., 2011)

| $m_{\infty}=\frac{1}{1+exp\left( \frac{-\left( V+39.57 \right)}{9.871} \right)}$ |  | | |
| --- | --- | --- | --- |
| $h_{\infty}=j_{\infty}=\frac{1}{1+exp\left( \frac{V+82.9}{6.086} \right)}$ |  | | |
| $h_{CaMK,\infty}=\frac{1}{1+exp\left( \frac{V+89.1}{6.086} \right)}$ |  | | |
| $\tau_{h, fast}=\frac{1}{1.432\cdot{10}^{-5}\cdot exp\left( \frac{-(V+1.196)}{6.285} \right)+6.149\cdot exp\left( \frac{V+0.5096}{20.27} \right)}$ | |  | |
| $\tau_{j}=2.038+\frac{1}{0.02136\cdot\exp\left( \frac{-(V+100.6)}{8.281} \right)+0.3052\cdot exp\left( \frac{V+0.9941}{38.45} \right)}$ | | |  |
| $G_{Na}=75, G_{NaL}=0.0075$ | | | |

Modified O’Hara et al. formulation

| $m_{\infty}=\frac{1}{1+exp\left( \frac{-\left( V+48.97 \right)}{7.5} \right)}$ | (Mora et al., 2017) | | |
| --- | --- | --- | --- |
| $h_{\infty}=j_{\infty}=\frac{1}{1+exp\left( \frac{V+78.5}{6.22} \right)}$ | (Passini et al., 2016) | | |
| $h_{CaMK,\infty}=\frac{1}{1+exp\left( \frac{V+84.7}{6.22} \right)}$ | (Passini et al., 2016) | | |
| $\tau_{h, fast}=\frac{1}{3.6860\cdot{10}^{-6}\cdot exp\left( \frac{-(V+3.8875)}{7.8579} \right)+16\cdot exp\left( \frac{V-0.4963}{9.1843} \right)}$ | | (Dutta et al., 2017) | |
| $\tau_{j}=4.8590+\frac{1}{0.8628\cdot\exp\left( \frac{-\left( V+116.7258 \right)}{7.6005} \right)+1.1096\cdot exp\left( \frac{V+6.2719}{9.0358} \right)}$ | | | (Dutta et al., 2017) |
| $G_{Na}=17 G_{NaL}=0.0144$ | | | |

Where

| $m_{\infty}$ | steady state activation gate of the sodium current (I_Na_) |
| --- | --- |
| $h_{\infty}$ | steady state inactivation gate of the I_Na_ |
| $j_{\infty}$ | recovery from inactivation for fast I_Na_ |
| $h_{CaMK,\infty}$ | phosphorylated steady state inactivation gate of the I_Na_ |
| $\tau_{h, fast}$ | fast time constant of gate h |
| $\tau_{j}$ | time constant of gate j |
| V | membrane voltage (mV) |
| $G_{Na}$ | I_Na_ conductance |
| $G_{NaL}$ | I_NaL_ conductance |

**3D ventricular model**

Cardiac DE-MRI was acquired by an MRI scanner Magnetom Avanto 1.5T (Siemens Healthcare, Erlangen, Germany) using a phased-array body surface coil, about 15 minutes after the administration of the gadolinium-based contrast MultiHance (gadobenate dimeglumine, 529 mg/ml) (Bracco Diagnostics Inc., Monroe Township, New Jersey, USA). The acquisition was synchronized with both ECG (ECG-gated) and breathing (navigator-gated), imaging the heart at the end-diastolic phase of cardiac cycle. The DE-MRI stack comprised 96 slices of 256×256 pixels encompassing the whole heart (ventricles and atria), with a pixel size of 1.4×1.4 mm and a slice thickness of 1.4 mm, thus resulting in isotropic voxel. The cardiac DE-MRI was acquired from the Hospital Clinic Universitari de Valencia (Valencia, Spain). Regarding the ethical considerations, the protocol was approved by the Ethics Committee for Clinical Research of the Hospital Clinic Universitari de Valencia, which certifies that the present study was conducted in accordance with the recommendations gathered in the Declaration of Helsinki, originally adopted by the General Assembly of the World Medical Association in 1964, and in its subsequent revisions. Furthermore, the patient, who underwent the standard clinical protocol, gave written informed consent for the use of his anonymized clinical data in this study.

We generated the 3D patient-specific bi-ventricular model by segmenting the short-axis slices from the cardiac DE-MRI using Seg3D software (Scientific Computing and Imaging Institute, University of Utah, USA) (SCII. Scientific Computing and Imaging Institute-University of Utah, 2016). We did it manually to perform a highly detailed segmentation of the whole ventricles, including papillary muscles and main endocardial trabecula (see Figure 1). An expert radiologist in cardiac imaging checked all segmentations in order to ensure the fidelity of the 3D reconstruction of the patient-specific anatomy. From the segmented DE-MRI stack, we generated a surface model of the ventricles, carefully checked with Blender (Blender Foundation, Amsterdam, The Netherlands) to refine and correct defects in the mesh at the local level after applying a global smoothing. Then, using the surface model as a template, we performed a volume meshing with MeshGems-Hexa (Distene S.A.S., Bruyeres-le-Chatel, France), obtaining a hexahedra-based volume mesh comprised by 4 million nodes (vertices) and 3.71 million elements, with an average edge length of 380 um.

**3D torso model**

The torso dataset was obtained from the online open repository at the Centre for Integrative Biomedical Computing (CBIC) from University of Utah (MacLeod et al., 1991). The whole torso MRI stack was acquired in the coronal plane with a slice thickness of 10 mm. We roughly segmented the main organs (lungs, liver, heart) and structures (bones, body contour, blood pools, great vessels) using Seg3D software. The resolution of the torso MRI hampered a detailed reconstruction of some important structures, so we used the reconstructed parts of the model as landmarks to fit a detailed torso model previously developed (Ferrer et al., 2015) by means of a linear transformation. Next, we replaced the ventricles in the fitted detailed torso model by our patient specific model and removed any intersections between our ventricular model and surrounding organs. Finally, we used TetGen (Si and Gärtner) to mesh the torso volume with tetrahedra, which resulted in 1.26 million nodes and 7.38 million elements organs. The average edge length was of 0.4 mm. Note that the problem of passive propagation of extracellular potentials, i.e. only diffusion without reaction component, does not require such a fine spatial resolution outside the heart domain (Prassl et al., 2009); for this reason, the mesh is highly refined only in the region of the ventricles.

We automatically labelled every tetrahedral element of the volume mesh as belonging to a given organ. The 3D torso model included bones, lungs, liver, whole heart (ventricles and atria) and blood pools of all cardiac chambers organs (see Figure 1). As in (Ferrer et al., 2015), conductivity values assigned to different organs and tissues were taken from the literature (Bradley et al., 2000; Bressler and Ding, 2006; Gabriel et al., 1996; Klepfer et al., 1997; Tun and Lachman, 2010). We considered isotropic propagation for all organs and tissues of our 3D torso model, except for the ventricular myocardium where we preserved the anisotropy imposed by the orientation of cardiac fibers. As in (Klepfer et al., 1997), for the space not covered by any organ or anatomical structure we set a conductivity of 0.239 296 S/m calculated as the average of the conductivities for the other tissues, including the skeletal muscle that was not considered as a specific region in our torso model. Finally, to simulate ECG signals we defined virtual electrodes on the surface of torso model corresponding to the precordial leads, which were placed in their standard positions (see Figure 1).

**Computational simulations**

To perform the simulations at the organ level, we used the software ELVIRA (Heidenreich et al., 2010), FEM solver specifically developed for solving the anisotropic reaction-diffusion equation of the monodomain model for cardiac EP (Roth, 1988). For the numerical solution of our simulations, we applied the conjugate gradient method with an integration time step of 0.02 ms, using implicit integration for the parabolic partial differential equation of monodomain model and explicit integration with adaptive time stepping for the systems of ordinary differential equations associated with the ionic model (O’hara et al., 2011).

To obtain ECG signals on the body surface, we used an approximation of the bidomain model (Geselowitz and Miller, 1983) to compute the extracellular potentials across the torso volume. This approximation, described elsewhere (Keller et al., 2010), comprises several steps. First, transmembrane potentials, previously computed by simulation at the organ level using the solver ELVIRA as explained above, were interpolated from the ventricular mesh model to the nodes of torso model corresponding to the ventricular myocardium. Then, solving the passive term (only diffusion) of the bidomain approach we obtained the extracellular potentials in the ventricles from the interpolated transmembrane voltages. Finally, applying Dirichlet boundary conditions at the ventricles-torso interface and Neumann-type conditions at the torso surface, the extracellular potentials were computed by using the FEM method to solve the Laplace equation over the volume mesh of the 3D torso model. To obtain the numerical solution of the problem, we used the conjugate gradient method with the incomplete Cholesky decomposition as a preconditioner.

# Supplementary Figures and Tables


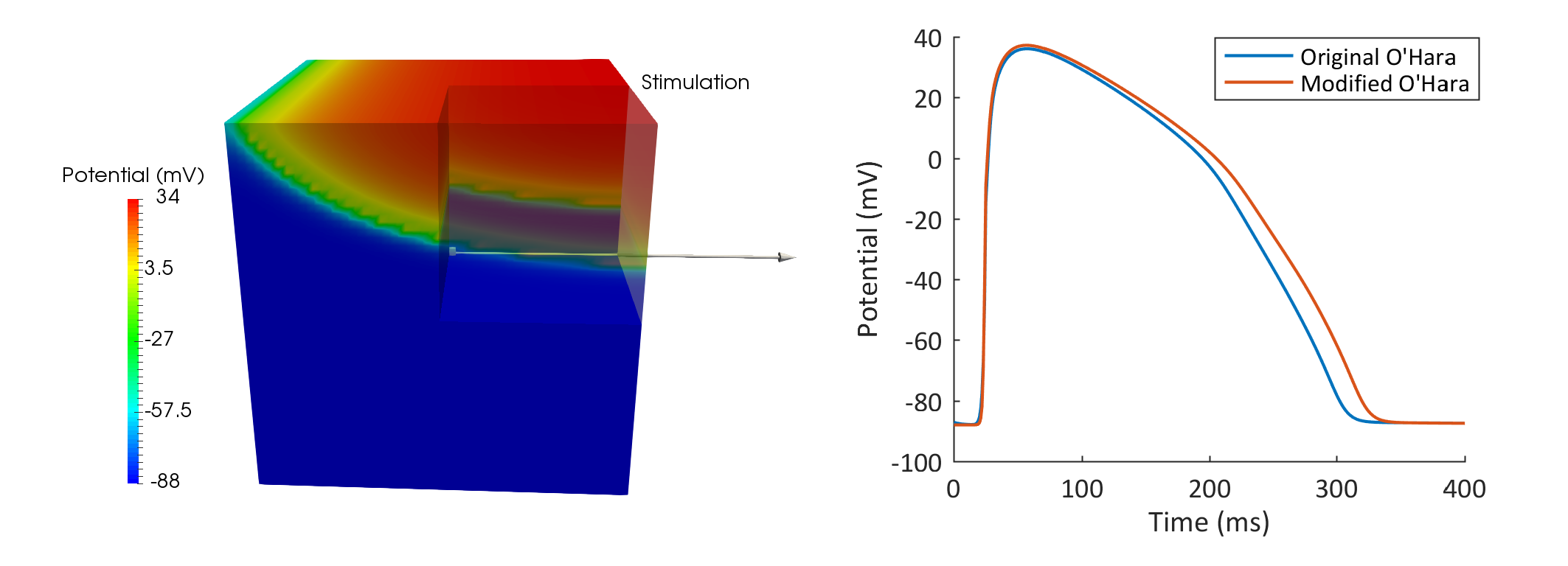


**Figure S1.** Action potentials simulated in a cube with original O’Hara model (blue line) and with modified O’Hara model (red line).


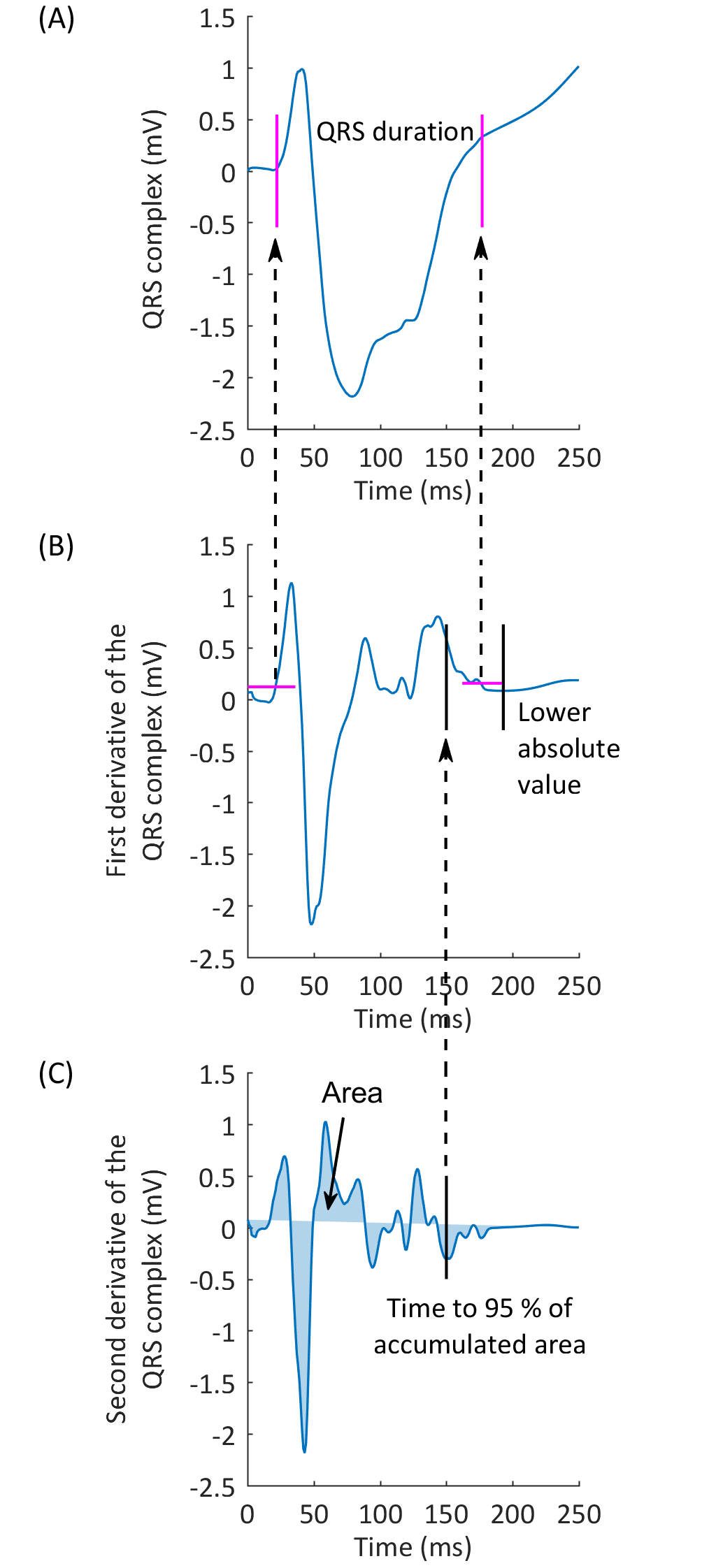


**Figure S2.** Measurement of QRS duration. **(A)** QRS Complex where the onset and end of the QRS is shown. **(B)** First derivative of the QRS complex with the thresholds used (magenta lines) to determinate the QRS duration. The black lines indicate the limits of the time interval. **(C)** Second derivative of the QRS complex. The blue region represents the area under of second derivative and the black line indicates the time to 95 % of accumulated area.


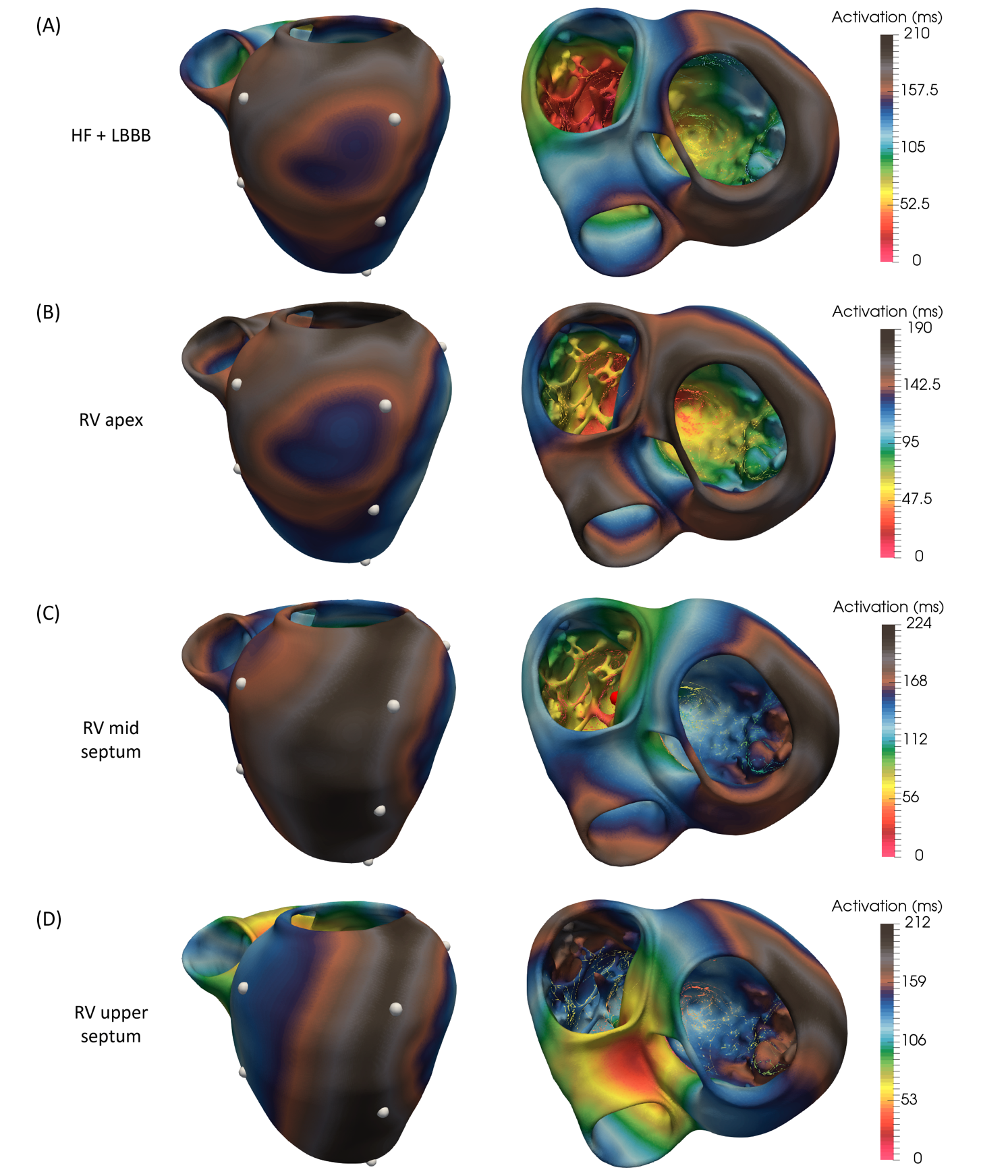


**Figure S3.** Activation maps. **(A)** HF + LBBB configuration showing LV anterior mid and basal regions as latest activated areas. **(B)** Univentricular stimulation with RV lead placed in the apex. **(C)** Univentricular stimulation with RV lead placed in the mid septum. **(D)** Univentricular stimulation with RV lead placed in upper septum.

**Table S1.** QRS duration (QRSd) estimated for all CRT configurations.

| **Lead locations** | | **QRS duration (ms)** | | | | | | | |
| --- | --- | --- | --- | --- | --- | --- | --- | --- | --- |
|  |  | **LV epicardial stimulation** | | | | **LV endocardial stimulation** | | | |
| **RV** | **LV** | **AVD = 100 VVD = 0** | **AVD = 100 VVD = 30** | **AVD = 140 VVD = 0** | **AVD = 140 VVD = 30** | **AVD = 100 VVD = 0** | **AVD = 100 VVD = 30** | **AVD = 140 VVD = 0** | **AVD = 140 VVD = 30** |
| Apex | Anterior - apex | 179 | 184 | 179 | 184 | 169 | 182 | 169 | 182 |
|  | Anterior - mid | 178 | 206 | 178 | 205 | 171 | 186 | 171 | 186 |
|  | Anterior - base | 181 | 201 | 181 | 201 | 175 | 187 | 175 | 186 |
|  | Lateral - apex | 181 | 196 | 181 | 195 | 169 | 175 | 169 | 174 |
|  | Lateral - mid | 175 | 206 | 175 | 206 | 171 | 189 | 171 | 188 |
|  | Lateral - base | 179 | 205 | 179 | 205 | 174 | 200 | 174 | 199 |
|  | Posterior - apex | 181 | 206 | 181 | 202 | 176 | 180 | 176 | 173 |
|  | Posterior - mid | 172 | 199 | 172 | 199 | 173 | 157 | 173 | 158 |
|  | Posterior - base | 173 | 194 | 173 | 194 | 171 | 194 | 171 | 193 |
| Mid Septum | Anterior - apex | 173 | 182 | 173 | 181 | 174 | 193 | 174 | 195 |
|  | Anterior - mid | 205 | 177 | 205 | 190 | 165 | 171 | 165 | 170 |
|  | Anterior - base | 182 | 189 | 182 | 194 | 177 | 174 | 177 | 178 |
|  | Lateral - apex | 182 | 205 | 182 | 203 | 171 | 188 | 171 | 188 |
|  | Lateral - mid | 174 | 203 | 174 | 198 | 175 | 201 | 175 | 193 |
|  | Lateral - base | 185 | 201 | 185 | 210 | 174 | 199 | 174 | 205 |
|  | Posterior - apex | 187 | 203 | 187 | 202 | 176 | 190 | 176 | 189 |
|  | Posterior - mid | 167 | 163 | 167 | 161 | 146 | 158 | 146 | 159 |
|  | Posterior - base | 181 | 190 | 181 | 206 | 182 | 196 | 182 | 201 |
| Upper Septum | Anterior - apex | 180 | 194 | 167 | 177 | 175 | 174 | 155 | 162 |
|  | Anterior - mid | 185 | 203 | 182 | 192 | 186 | 187 | 165 | 165 |
|  | Anterior - base | 183 | 194 | 188 | 192 | 178 | 185 | 176 | 177 |
|  | Lateral - apex | 185 | 200 | 177 | 194 | 166 | 162 | 156 | 157 |
|  | Lateral - mid | 181 | 182 | 171 | 167 | 183 | 196 | 157 | 154 |
|  | Lateral - base | 178 | 196 | 170 | 171 | 180 | 191 | 172 | 175 |
|  | Posterior - apex | 198 | 205 | 183 | 206 | 171 | 167 | 168 | 166 |
|  | Posterior - mid | 159 | 162 | 155 | 149 | 158 | 154 | 143 | 140 |
|  | Posterior - base | 180 | 180 | 190 | 190 | 183 | 176 | 187 | 185 |

**Table S2.** Total activation time (TAT) values estimated for all CRT configurations.

| **Lead locations** | | **Total activation time (ms)** | | | | | | | |
| --- | --- | --- | --- | --- | --- | --- | --- | --- | --- |
|  |  | **LV epicardial stimulation** | | | | **LV endocardial stimulation** | | | |
| **RV** | **LV** | **AVD = 100 VVD = 0** | **AVD = 100 VVD = 30** | **AVD = 140 VVD = 0** | **AVD = 140 VVD = 30** | **AVD = 100 VVD = 0** | **AVD = 100 VVD = 30** | **AVD = 140 VVD = 0** | **AVD = 140 VVD = 30** |
| Apex | Anterior - apex | 190 | 218 | 190 | 218 | 185 | 202 | 185 | 202 |
|  | Anterior - mid | 190 | 218 | 190 | 218 | 190 | 209 | 190 | 209 |
|  | Anterior - base | 185 | 205 | 185 | 205 | 178 | 187 | 178 | 187 |
|  | Lateral - apex | 190 | 220 | 190 | 220 | 175 | 200 | 175 | 200 |
|  | Lateral - mid | 190 | 220 | 190 | 220 | 190 | 218 | 190 | 218 |
|  | Lateral - base | 188 | 209 | 188 | 209 | 190 | 213 | 190 | 213 |
|  | Posterior - apex | 190 | 219 | 190 | 219 | 181 | 202 | 181 | 202 |
|  | Posterior - mid | 178 | 202 | 178 | 202 | 174 | 181 | 174 | 181 |
|  | Posterior - base | 181 | 204 | 181 | 204 | 181 | 204 | 181 | 203 |
| Mid Septum | Anterior - apex | 203 | 212 | 203 | 212 | 185 | 206 | 185 | 204 |
|  | Anterior - mid | 212 | 224 | 212 | 224 | 195 | 210 | 195 | 210 |
|  | Anterior - base | 194 | 207 | 194 | 207 | 186 | 188 | 186 | 187 |
|  | Lateral - apex | 215 | 228 | 215 | 228 | 185 | 200 | 185 | 200 |
|  | Lateral - mid | 187 | 215 | 187 | 211 | 186 | 214 | 186 | 210 |
|  | Lateral - base | 186 | 215 | 186 | 211 | 186 | 215 | 186 | 211 |
|  | Posterior - apex | 218 | 220 | 218 | 220 | 185 | 206 | 185 | 203 |
|  | Posterior - mid | 185 | 205 | 185 | 203 | 179 | 182 | 179 | 182 |
|  | Posterior - base | 191 | 215 | 191 | 210 | 190 | 214 | 190 | 210 |
| Upper Septum | Anterior - apex | 200 | 212 | 200 | 212 | 180 | 185 | 180 | 180 |
|  | Anterior - mid | 209 | 222 | 209 | 222 | 192 | 210 | 192 | 206 |
|  | Anterior - base | 192 | 210 | 192 | 205 | 187 | 197 | 185 | 186 |
|  | Lateral - apex | 211 | 225 | 211 | 225 | 171 | 175 | 171 | 171 |
|  | Lateral - mid | 189 | 210 | 184 | 192 | 188 | 203 | 176 | 188 |
|  | Lateral - base | 189 | 210 | 175 | 189 | 189 | 210 | 175 | 189 |
|  | Posterior - apex | 212 | 220 | 212 | 220 | 181 | 182 | 181 | 181 |
|  | Posterior - mid | 170 | 178 | 170 | 171 | 158 | 169 | 152 | 164 |
|  | Posterior - base | 190 | 190 | 190 | 190 | 188 | 188 | 188 | 188 |

**Table S3.** Time to 90 % of ventricular activation (t_90_) estimated for all CRT configurations.

| **Lead locations** | | **Time to 90 % of ventricular activation (ms)** | | | | | | | |
| --- | --- | --- | --- | --- | --- | --- | --- | --- | --- |
|  |  | **LV epicardial stimulation** | | | | **LV endocardial stimulation** | | | |
| **RV** | **LV** | **AVD = 100 VVD = 0** | **AVD = 100 VVD = 30** | **AVD = 140 VVD = 0** | **AVD = 140 VVD = 30** | **AVD = 100 VVD = 0** | **AVD = 100 VVD = 30** | **AVD = 140 VVD = 0** | **AVD = 140 VVD = 30** |
| Apex | Anterior - apex | 145 | 165 | 145 | 165 | 136 | 141 | 136 | 141 |
|  | Anterior - mid | 136 | 155 | 136 | 155 | 134 | 150 | 134 | 149 |
|  | Anterior - base | 131 | 150 | 131 | 150 | 129 | 145 | 129 | 145 |
|  | Lateral - apex | 143 | 170 | 143 | 169 | 129 | 134 | 129 | 134 |
|  | Lateral - mid | 136 | 156 | 136 | 156 | 139 | 156 | 139 | 156 |
|  | Lateral - base | 133 | 153 | 133 | 153 | 133 | 155 | 133 | 155 |
|  | Posterior - apex | 145 | 172 | 145 | 172 | 139 | 143 | 139 | 143 |
|  | Posterior - mid | 135 | 147 | 135 | 147 | 122 | 130 | 122 | 130 |
|  | Posterior - base | 137 | 158 | 137 | 158 | 137 | 157 | 137 | 157 |
| Mid Septum | Anterior - apex | 152 | 161 | 152 | 161 | 133 | 142 | 133 | 141 |
|  | Anterior - mid | 144 | 153 | 144 | 152 | 134 | 145 | 134 | 144 |
|  | Anterior - base | 153 | 164 | 153 | 163 | 150 | 154 | 150 | 154 |
|  | Lateral - apex | 155 | 173 | 155 | 172 | 128 | 135 | 128 | 134 |
|  | Lateral - mid | 138 | 154 | 138 | 153 | 139 | 155 | 139 | 154 |
|  | Lateral - base | 143 | 155 | 143 | 154 | 145 | 158 | 145 | 157 |
|  | Posterior - apex | 161 | 172 | 161 | 172 | 138 | 144 | 138 | 143 |
|  | Posterior - mid | 145 | 150 | 145 | 149 | 127 | 131 | 127 | 130 |
|  | Posterior - base | 162 | 169 | 162 | 169 | 162 | 168 | 162 | 168 |
| Upper Septum | Anterior - apex | 147 | 157 | 143 | 153 | 127 | 137 | 125 | 134 |
|  | Anterior - mid | 151 | 160 | 144 | 153 | 141 | 151 | 134 | 143 |
|  | Anterior - base | 157 | 168 | 153 | 162 | 153 | 156 | 150 | 153 |
|  | Lateral - apex | 151 | 167 | 145 | 162 | 123 | 130 | 122 | 129 |
|  | Lateral - mid | 142 | 153 | 133 | 142 | 136 | 148 | 128 | 139 |
|  | Lateral - base | 147 | 155 | 139 | 146 | 148 | 158 | 141 | 149 |
|  | Posterior - apex | 151 | 162 | 149 | 161 | 132 | 140 | 132 | 138 |
|  | Posterior - mid | 142 | 147 | 140 | 145 | 127 | 132 | 123 | 128 |
|  | Posterior - base | 154 | 163 | 152 | 161 | 153 | 162 | 151 | 160 |

**Table S4.** QRS area estimated for all CRT configurations.

| **Lead locations** | | **QRS area (mV·ms)** | | | | | | | |
| --- | --- | --- | --- | --- | --- | --- | --- | --- | --- |
|  |  | **LV epicardial stimulation** | | | | **LV endocardial stimulation** | | | |
| **RV** | **LV** | **AVD = 100 VVD = 0** | **AVD = 100 VVD = 30** | **AVD = 140 VVD = 0** | **AVD = 140 VVD = 30** | **AVD = 100 VVD = 0** | **AVD = 100 VVD = 30** | **AVD = 140 VVD = 0** | **AVD = 140 VVD = 30** |
| Apex | Anterior - apex | 157.2 | 156.0 | 157.2 | 156.0 | 146.1 | 125.1 | 146.1 | 124.3 |
|  | Anterior - mid | 108.4 | 82.7 | 108.4 | 82.8 | 115.7 | 95.6 | 115.7 | 95.1 |
|  | Anterior - base | 103.5 | 87.3 | 103.5 | 87.8 | 101.6 | 73.1 | 101.6 | 73.2 |
|  | Lateral - apex | 115.5 | 134.3 | 115.5 | 133.6 | 116.7 | 116.2 | 116.7 | 115.6 |
|  | Lateral - mid | 84.3 | 78.7 | 84.3 | 78.1 | 130.1 | 118.7 | 130.1 | 117.4 |
|  | Lateral - base | 119.2 | 109.0 | 119.2 | 109.3 | 125.9 | 110.1 | 125.9 | 110.3 |
|  | Posterior - apex | 125.9 | 109.7 | 125.9 | 108.9 | 117.2 | 109.5 | 117.2 | 108.4 |
|  | Posterior - mid | 112.6 | 79.1 | 112.6 | 79.1 | 86.9 | 81.8 | 86.9 | 82.0 |
|  | Posterior - base | 131.1 | 113.5 | 131.1 | 114.0 | 129.0 | 109.3 | 129.0 | 109.5 |
| Mid Septum | Anterior - apex | 119.8 | 124.2 | 119.8 | 127.1 | 102.1 | 115.4 | 102.1 | 113.9 |
|  | Anterior - mid | 76.6 | 64.7 | 76.6 | 58.6 | 92.8 | 72.5 | 92.8 | 78.6 |
|  | Anterior - base | 101.8 | 97.8 | 101.8 | 87.6 | 110.0 | 108.0 | 110.0 | 100.1 |
|  | Lateral - apex | 96.8 | 115.7 | 96.8 | 108.8 | 99.6 | 109.9 | 99.6 | 108.9 |
|  | Lateral - mid | 61.0 | 94.0 | 61.0 | 84.6 | 104.1 | 111.3 | 104.1 | 109.0 |
|  | Lateral - base | 105.7 | 87.9 | 105.7 | 83.6 | 111.6 | 91.2 | 111.6 | 87.6 |
|  | Posterior - apex | 84.7 | 100.0 | 84.7 | 98.0 | 109.5 | 102.3 | 109.5 | 101.1 |
|  | Posterior - mid | 98.1 | 94.3 | 98.1 | 91.3 | 95.7 | 89.0 | 95.7 | 90.5 |
|  | Posterior - base | 127.9 | 103.8 | 127.9 | 97.5 | 125.1 | 103.7 | 125.1 | 96.2 |
| Upper Septum | Anterior - apex | 91.7 | 118.4 | 94.9 | 118.6 | 93.8 | 103.3 | 87.9 | 96.1 |
|  | Anterior - mid | 65.5 | 68.1 | 69.0 | 64.0 | 73.1 | 70.7 | 86.5 | 80.4 |
|  | Anterior - base | 114.5 | 118.4 | 113.9 | 104.9 | 123.8 | 128.7 | 118.9 | 106.9 |
|  | Lateral - apex | 86.6 | 110.6 | 82.4 | 104.6 | 96.6 | 100.5 | 93.9 | 96.7 |
|  | Lateral - mid | 93.7 | 103.1 | 74.3 | 73.4 | 95.0 | 104.2 | 88.0 | 90.6 |
|  | Lateral - base | 106.5 | 98.7 | 107.0 | 97.9 | 110.0 | 96.3 | 112.6 | 102.9 |
|  | Posterior - apex | 75.9 | 91.8 | 78.2 | 90.3 | 96.0 | 95.8 | 97.5 | 93.3 |
|  | Posterior - mid | 102.0 | 93.5 | 106.7 | 98.0 | 92.8 | 90.8 | 97.7 | 90.2 |
|  | Posterior - base | 116.0 | 111.3 | 119.5 | 111.7 | 115.8 | 111.5 | 118.6 | 109.6 |

**Table S5.** Time to reach the 90% of the QRS area (t_90_QRSa) estimated for all CRT configurations.

| **Lead locations** | | **Time to 90 % QRS area (ms)** | | | | | | | |
| --- | --- | --- | --- | --- | --- | --- | --- | --- | --- |
|  |  | **LV epicardial stimulation** | | | | **LV endocardial stimulation** | | | |
| **RV** | **LV** | **AVD = 100 VVD = 0** | **AVD = 100 VVD = 30** | **AVD = 140 VVD = 0** | **AVD = 140 VVD = 30** | **AVD = 100 VVD = 0** | **AVD = 100 VVD = 30** | **AVD = 140 VVD = 0** | **AVD = 140 VVD = 30** |
| Apex | Anterior - apex | 142 | 156 | 142 | 156 | 131 | 140 | 131 | 140 |
|  | Anterior - mid | 138 | 160 | 138 | 160 | 133 | 149 | 133 | 149 |
|  | Anterior - base | 127 | 150 | 127 | 149 | 125 | 148 | 125 | 148 |
|  | Lateral - apex | 144 | 164 | 144 | 163 | 129 | 136 | 129 | 135 |
|  | Lateral - mid | 141 | 165 | 141 | 165 | 138 | 158 | 138 | 158 |
|  | Lateral - base | 133 | 154 | 133 | 153 | 133 | 156 | 133 | 156 |
|  | Posterior - apex | 144 | 173 | 144 | 172 | 138 | 144 | 138 | 143 |
|  | Posterior - mid | 135 | 154 | 135 | 154 | 128 | 135 | 128 | 135 |
|  | Posterior - base | 138 | 162 | 138 | 162 | 138 | 162 | 138 | 162 |
| Mid Septum | Anterior - apex | 150 | 156 | 150 | 155 | 136 | 144 | 136 | 144 |
|  | Anterior - mid | 154 | 159 | 154 | 159 | 138 | 149 | 138 | 147 |
|  | Anterior - base | 160 | 173 | 160 | 171 | 160 | 162 | 160 | 163 |
|  | Lateral - apex | 155 | 175 | 155 | 173 | 131 | 141 | 131 | 140 |
|  | Lateral - mid | 153 | 172 | 153 | 170 | 148 | 162 | 148 | 161 |
|  | Lateral - base | 149 | 167 | 149 | 163 | 151 | 169 | 151 | 166 |
|  | Posterior - apex | 164 | 177 | 164 | 176 | 139 | 149 | 139 | 147 |
|  | Posterior - mid | 150 | 153 | 150 | 153 | 130 | 137 | 130 | 135 |
|  | Posterior - base | 168 | 176 | 168 | 176 | 167 | 175 | 167 | 174 |
| Upper Septum | Anterior - apex | 143 | 156 | 139 | 149 | 131 | 138 | 127 | 134 |
|  | Anterior - mid | 153 | 166 | 148 | 159 | 147 | 157 | 136 | 146 |
|  | Anterior - base | 161 | 173 | 160 | 169 | 158 | 162 | 159 | 162 |
|  | Lateral - apex | 148 | 165 | 147 | 162 | 128 | 133 | 125 | 130 |
|  | Lateral - mid | 143 | 153 | 134 | 147 | 141 | 153 | 132 | 145 |
|  | Lateral - base | 150 | 153 | 148 | 150 | 153 | 156 | 151 | 152 |
|  | Posterior - apex | 153 | 168 | 150 | 166 | 136 | 141 | 134 | 139 |
|  | Posterior - mid | 144 | 150 | 144 | 150 | 130 | 137 | 128 | 131 |
|  | Posterior - base | 164 | 171 | 163 | 169 | 163 | 169 | 162 | 168 |

# Supplementary Videos

Video 1_CRT

Video 2_RETROGRADE

# Supplementary references

Bradley, C. P., Pullan, A. J., and Hunter, P. J. (2000). Effects of Material Properties and Geometry on Electrocardiographic Forward Simulations. *Ann. Biomed. Eng.* 28, 721–741. doi:10.1114/1.1289467.

Bressler, S. L., and Ding, M. (2006). “Event-Related Potentials,” in *Wiley Encyclopedia of Biomedical Engineering* (Hoboken, NJ, USA: John Wiley & Sons, Inc.). doi:10.1002/9780471740360.ebs0455.

Dutta, S., Mincholé, A., Quinn, T. A., and Rodriguez, B. (2017). Electrophysiological properties of computational human ventricular cell action potential models under acute ischemic conditions. *Prog. Biophys. Mol. Biol.* doi:10.1016/j.pbiomolbio.2017.02.007.

Ferrer, A., Sebastián, R., Sánchez-Quintana, D., Rodríguez, J. F., Godoy, E. J., Martínez, L., et al. (2015). Detailed anatomical and electrophysiological models of human atria and torso for the simulation of atrial activation. *PLoS One*. doi:10.1371/journal.pone.0141573.

Gabriel, S., Lau, R. W., and Gabriel, C. (1996). The dielectric properties of biological tissues: II. Measurements in the frequency range 10 Hz to 20 GHz. *Phys. Med. Biol.* 41, 2251–2269. doi:10.1088/0031-9155/41/11/002.

Geselowitz, D. B., and Miller, W. T. (1983). A bidomain model for anisotropic cardiac muscle. *Ann. Biomed. Eng.* 11, 191–206. doi:10.1007/BF02363286.

Heidenreich, E. A., Ferrero, J. M., Doblaré, M., and Rodríguez, J. F. (2010). Adaptive macro finite elements for the numerical solution of monodomain equations in cardiac electrophysiology. *Ann. Biomed. Eng.* doi:10.1007/s10439-010-9997-2.

Keller, D. U. J., Weber, F. M., Seemann, G., and Dössel, O. (2010). Ranking the influence of tissue conductivities on forward-calculated ecgs. *IEEE Trans. Biomed. Eng.* doi:10.1109/TBME.2010.2046485.

Klepfer, R. N., Johnson, C. R., and Macleod, R. S. (1997). The effects of inhomogeneities and anisotropies on electrocardiographic fields: a 3-D finite-element study. *IEEE Trans. Biomed. Eng.* 44, 706–719. doi:10.1109/10.605427.

MacLeod, R. S., Johnson, C. R., and Ershler, P. R. (1991). Construction of an inhomogeneous model of the human torso for use in computational electrocardiography. *Proc. Annu. Conf. Eng. Med. Biol.* 13, 688–689.

Mora, M. T., Ferrero, J. M., Romero, L., and Trenor, B. (2017). Sensitivity analysis revealing the effect of modulating ionic mechanisms on calcium dynamics in simulated human heart failure. *PLoS One*. doi:10.1371/journal.pone.0187739.

O’hara, T., Virág, L., Varró, A., and Rudy, Y. (2011). Simulation of the Undiseased Human Cardiac Ventricular Action Potential: Model Formulation and Experimental Validation. *PLoS Comput Biol* 7, 1002061. doi:10.1371/journal.pcbi.1002061TA´MOP-4.2.2-08/1-2008-0013.

Passini, E., Mincholé, A., Coppini, R., Cerbai, E., Rodriguez, B., Severi, S., et al. (2016). Mechanisms of pro-arrhythmic abnormalities in ventricular repolarisation and anti-arrhythmic therapies in human hypertrophic cardiomyopathy. *J. Mol. Cell. Cardiol.* doi:10.1016/j.yjmcc.2015.09.003.

Prassl, A. J., Kickinger, F., Ahammer, H., Grau, V., Schneider, J. E., Hofer, E., et al. (2009). Automatically generated, anatomically accurate meshes for cardiac electrophysiology problems. *IEEE Trans. Biomed. Eng.* 56, 1318–30. doi:10.1109/TBME.2009.2014243.

Roth, B. J. (1988). The electrical potential produced by a strand of cardiac muscle: A bidomain analysis. *Ann. Biomed. Eng.* 16, 609–637. doi:10.1007/BF02368018.

SCII. Scientific Computing and Imaging Institute-University of Utah (2016). Seg3D. Volumetric image segmentation and visualization.

Si, H., and Gärtner, K. “Meshing Piecewise Linear Complexes by Constrained Delaunay Tetrahedralizations,” in *Proceedings of the 14th International Meshing Roundtable* (Berlin/Heidelberg: Springer-Verlag), 147–163. doi:10.1007/3-540-29090-7_9.

Tun, P. A., and Lachman, M. E. (2010). The association between computer use and cognition across adulthood: Use it so you won’t lose it? *Psychol. Aging* 25, 560–568. doi:10.1037/a0019543.
